# Notch-Deficient Skin Induces a Lethal Systemic B-Lymphoproliferative Disorder by Secreting TSLP, a Sentinel for Epidermal Integrity

**DOI:** 10.1371/journal.pbio.0060123

**Published:** 2008-05-27

**Authors:** Shadmehr Demehri, Zhenyi Liu, Jonghyeob Lee, Meei-Hua Lin, Seth D Crosby, Christopher J Roberts, Perry W Grigsby, Jeffrey H Miner, Andrew G Farr, Raphael Kopan

**Affiliations:** 1 Department of Developmental Biology, Washington University School of Medicine, St. Louis, Missouri, United States of America; 2 Division of Dermatology, Washington University School of Medicine, St. Louis, Missouri, United States of America; 3 Renal Division, Department of Medicine, Washington University School of Medicine, St. Louis, Missouri, United States of America; 4 Genome Sequencing Center, Washington University School of Medicine, St. Louis, Missouri, United States of America; 5 Rosetta Inpharmatics, Seattle, Washington, United States of America; 6 Department of Radiation Oncology, Washington University School of Medicine, St. Louis, Missouri, United States of America; 7 Department of Biological Structure and Department of Immunology, University of Washington, Seattle, Washington, United States of America; Massachusetts Institute of Technology, United States of America

## Abstract

Epidermal keratinocytes form a highly organized stratified epithelium and sustain a competent barrier function together with dermal and hematopoietic cells. The Notch signaling pathway is a critical regulator of epidermal integrity. Here, we show that keratinocyte-specific deletion of total Notch signaling triggered a severe systemic B-lymphoproliferative disorder, causing death. RBP-j is the DNA binding partner of Notch, but both RBP-j–dependent and independent Notch signaling were necessary for proper epidermal differentiation and lipid deposition. Loss of both pathways caused a persistent defect in skin differentiation/barrier formation. In response, high levels of thymic stromal lymphopoietin (TSLP) were released into systemic circulation by Notch-deficient keratinocytes that failed to differentiate, starting in utero. Exposure to high TSLP levels during neonatal hematopoiesis resulted in drastic expansion of peripheral pre- and immature B-lymphocytes, causing B-lymphoproliferative disorder associated with major organ infiltration and subsequent death, a previously unappreciated systemic effect of TSLP. These observations demonstrate that local skin perturbations can drive a lethal systemic disease and have important implications for a wide range of humoral and autoimmune diseases with skin manifestations.

## Author Summary

Skin is the largest organ of the body, forming an elaborate barrier that prevents water loss and protects the internal environment from outside invaders. When this barrier is compromised, keratinocytes, keratin-producing epidermal cells, alert and recruit the immune cells to the site of the breach as part of an adaptive defense mechanism. However, chronic activation of such an “alarm” could have undesired consequences. Using genetic engineering to progressively remove components of Notch signaling from mouse skin in utero resulted in chronic skin-barrier defects, mimicking a form of human skin disease called atopic dermatitis. Surprisingly, we discovered that a persistent alarm signal in newborns triggered a systemic B-lymphoproliferative disorder, which precisely mirrored the degree of skin defect and was lethal in its extreme form. This alarm signal, in the form of a cytokine called thymic stromal lymphopoietin, was produced by Notch-deficient keratinocytes that failed to form a competent skin barrier. Therefore, we uncovered a long-range proliferative effect on fetal pre-B cells in vivo that is induced by injured skin and mediated by thymic stromal lymphopoietin. These findings highlight the central role that skin-derived factors can play in initiating systemic diseases with skin involvement.

## Abstract 3

A skin-derived factor plays a lead role in initiating a lethal systemic disease. The current findings point to a novel impact of Notch-deficient skin on hematopoiesis, where unique biology of a keratinocyte-derived cytokine contributes to a severe neonatal blood disease.

## Introduction

The vertebrate skin is an organ in which keratinocytes, underlying mesenchymal cells, and circulating hematopoietic cells engage in reciprocal communication as they monitor organ integrity [1]. Therefore, skin is an ideal system in which to study how complex, multicompartmental networks function. Epidermal keratinocytes are organized in several distinct layers with the innermost (basal) layer containing the stem cells and transiently amplifying cells [2]. The next layer contains spinous cells that, under normal conditions, begin a terminal differentiation program, giving rise to granular and cornified layers [3,4]. However, after injury, spinous cells proliferate to contribute to the restoration of an intact integument and produce cytokines that trigger an inflammatory response [1]. Defects in execution of this terminal differentiation program can lead to what are collectively known as skin-barrier defects [5].

Notch activation contributes to spinous cell differentiation. Loss of canonical Notch signaling, induced by deletion of the *RBPSUH* gene (coding for RBP-j, the DNA binding partner of Notch), causes severe epidermal barrier and differentiation defects highlighted by reduced spinous, granular, and cornified layer cells [6]. However, overexpression of activated Notch1 exclusively in basal cells under the *K14* promoter triggers their premature differentiation into spinous cells, concomitant with loss of proliferative capacity [6]. The pattern of Notch1 activation in epidermal keratinocytes is consistent with its proposed role in suppressing basal cell proliferation and promoting spinous cell differentiation via cell autonomous modulation of targets [6–11]. However, overexpression of activated Notch1 in the differentiated spinous cells (where it is normally present) triggers basal cell hyperproliferation and formation of acanthotic epidermis with a thickened granular layer [12], indicating that the Notch signaling pathway has a complex role by not only promoting differentiation and exit from the basal layer but also by contributing in a non-cell autonomous fashion to skin homeostasis [12,13]. How Notch performs its functions within spinous cells is a matter of some controversy, and multiple autonomous mechanisms have been proposed, including both canonical and noncanonical pathways [6–11].

**Figure 1:**
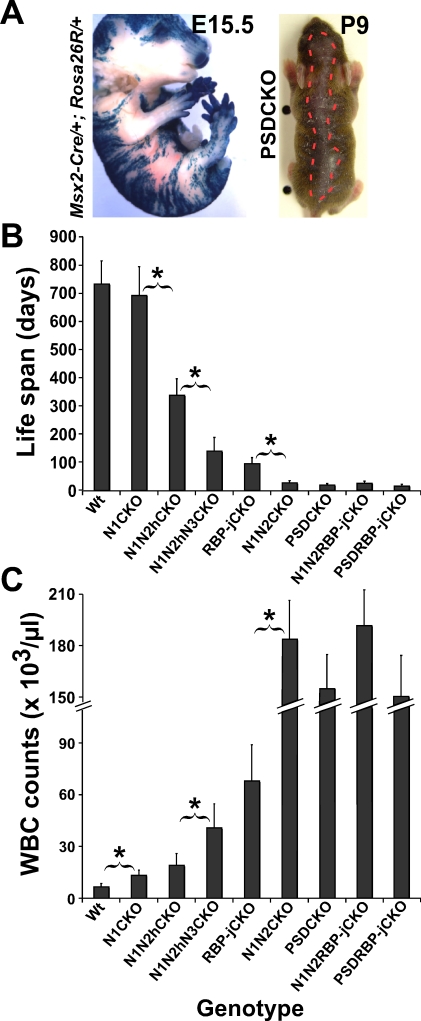
Notch Dosage in the Skin, the Animals' Life Span, and WBC Count Are Tightly Correlated (A) Chimeric pattern of *Msx2*-*Cre* activity is shown on the left by X-gal staining of an E15.5 *Msx2*-*Cre*/+; *Rosa26R*/+ embryo (also see [14,15]). On the right, the dorsal patch of hair loss in P9 *Msx2*-*Cre*/+; *PS1^flox^* ^/*flox*^; *PS2* ^–/–^ (PSDCKO) mice (border is highlighted by red broken line) matches the area of *Cre* activity. Progressive (B) shortening of life span and (C) increase in peak WBC count measured during the second week of life are caused by stepwise removal of Notch proteins, RBP-j, Presenilins, or certain combinations of those. Abbreviations: wild type (Wt), *Msx2*-*Cre*/+; *N1^flox^* ^/*flox*^ (N1CKO), *Msx2*-*Cre*/+; *N1^flox^* ^/*flox*^; *N2^flox^* ^/+^ (N1N2hCKO), *Msx2*-*Cre*/+; *N1^flox^* ^/*flox*^; *N2^flox^* ^/+^; *N3* ^–/–^ (N1N2hN3CKO), *Msx2*-*Cre*/+; *N1^flox^* ^/*flox*^; *N2^flox^* ^/*flox*^ (N1N2CKO), PSDCKO, *Msx2*-*Cre*/+; *RBP-j^flox^* ^/*flox*^ (RBP-jCKO), *Msx2*-*Cre*/+; *PS1^flox^* ^/*flox*^; *PS2* ^–/–^; *RBP-j^flox^* ^/*flox*^; (PSDRBP-jCKO), and *Msx2*-*Cre*/+; *N1^flox^* ^/*flox*^; *N2^flox^* ^/*flox*^; *RBP-j^flox^* ^/*flox*^ (N1N2RBP-jCKO). Note that N1N2CKO, PSDCKO, PSDRBP-jCKO, and N1N2RBP-jCKO animals live only a few weeks and experience severe leukocytosis. Data are collected from lifelong monitoring of 10 to 20 animals from each genotype. Significant difference (*p* < 0.05) between adjacent genotypes is highlighted by an asterisk.

To study the role of Notch signaling in skin homeostasis and barrier formation, we used the *Msx2-Cre* line to delete components of the Notch signaling pathway in skin keratinocytes [14]. A burst of *Msx2-Cre* expression on embryonic day 9.5 (E9.5) creates chimeric skin with dorsal and ventral patches (clones) lacking floxed alleles, confining the consequences of gene loss only to a fraction of the surface area (for a detailed analysis of *Msx2-Cre* expression in skin, see [14,15]). This allows animals lacking total Notch signaling to survive through birth (Figure 1A). With this system, we have shown previously that Notch loss also involves non-cell autonomous alteration in transforming growth factor ß and insulin-like growth factor signaling [15]. Removal of both Notch1 and Notch2 proteins or both presenilin-1 (PS1) and presenilin-2 (PS2) proteins (the catalytic subunits of γ-secretase) within epidermal clones is sufficient to cause early postnatal lethality [14]. In the current study, we investigated the mechanistic basis for this early demise.

The progressive loss of Notch alleles in skin keratinocytes generated a dose-dependent increase in *thymic stromal lymphopoietin* (*TSLP*) expression by suprabasal keratinocytes in direct response to defective skin differentiation/barrier formation in utero. TSLP is a recently discovered, epidermally derived cytokine implicated in the pathogenesis of atopic dermatitis and asthma [16]. Like interleukin 7 (IL-7), TSLP can support B cell development; however, the in vivo effects of high TSLP levels on B-lymphopoiesis are not fully understood [17]. A perinatal increase in TSLP levels produced a dose-dependent expansion of pre- and immature B cells in the periphery, causing a B-lymphoproliferative disorder (B-LPD), a previously unappreciated effect of TSLP. In its extreme form, B-LPD complications, including B cell infiltration into vital organs, culminated in death. Therefore, this study provides the first physiological confirmation that skin perturbation can cause a lethal systemic disease.

## Results

### Mice Lacking γ-Secretase or All Notch Proteins in the Skin Develop Severe B-LPD after Birth

Removal of total Notch signaling from the skin using *Msx2-Cre* led to death at weaning (Figure 1B; see [14]). To identify the cause of death in *Msx2*-*Cre*/+; *N1^flox^* ^/*flox*^; *N2^flox^* ^/*flox*^ (N1N2CKO) and *Msx2*-*Cre*/+; *PS1^flox^* ^/*flox*^; *PS2* ^–/–^ (PSDCKO) mice, a comprehensive necropsy was performed on the moribund animals. Surprisingly, we found that mice from both genotypes had extremely high white blood cell (WBC) counts (>150,000 cells/μl) at around the time of death (Figure 1C). Importantly, analysis of an extensive Notch allelic series revealed that the severity of the leukocytosis during the first few weeks of life was correlated inversely with Notch dosage (Figure 1C).

**Figure 2:**
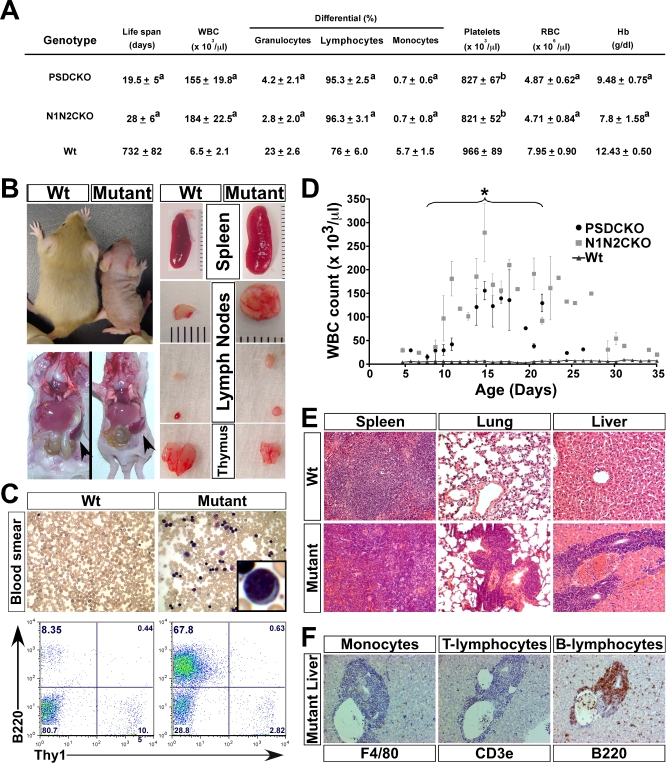
Mice Lacking Total Notch Signaling in the Skin Develop Severe B-LPD (A) Table shows the peripheral blood analysis of the mutant mice during the second week of life when WBC counts are at their maximum (*n* = 6, for each group). The values are presented as mean ± standard deviation (“a” indicates *p* < 0.001 and “b” indicates *p* < 0.05 (compared to wild-type control)). (B) Macroscopic examination of a P14 mutant (N1N2CKO) compared to its wild-type littermate reveals the mutant's smaller body size yet significantly larger spleen and lymph nodes. (C) Peripheral blood smear (Giemsa-stained; 250× magnification) and FC analysis show the appearance of lymphoblasts (inset) and the expansion of B220^+^ B cells in the mutant blood, respectively. (D) Monitoring the mutant animals' WBC counts over their life span (*n* = 10 for each genotype) demonstrates a surge during the first 2 weeks and plateau during the third week of life, when most mutants die (asterisk). Note the trend of WBC counts toward normalization in a few mice that live a few days longer. Severe B-LPD leads to infiltration of liver, lung, and spleen of the mutant animals with B220^+^ B cells shown on (E) hematoxylin-and-eosin-stained tissue sections and (F) antibody-stained liver sections (200× magnification).

To characterize the cells causing leukocytosis, we analyzed various hematopoietic parameters in Notch-deficient mice. Peripheral blood analysis showed that the leukocytosis in both N1N2CKO and PSDCKO animals was of a lymphoblastic/lymphocytic nature; hence the mice displayed a severe case of lymphoproliferative disorder (LPD; Figure 2A). This LPD was accompanied by the failure of bone marrow (BM) to participate in normal hematopoiesis, as demonstrated by reduced red blood cells and platelets (normocytic anemia and thrombocytopenia; Figure 2A). Because the blood phenotypes of N1N2CKO and PSDCKO mice were similar, we use the term “mutant” to describe the common LPD features of both genotypes and “wild type” for all genetic combinations that either lack *Cre* or carry *Msx2-Cre* and a wild-type allele of *PS1*. Macroscopic examination showed that mutant mice were smaller than their wild-type littermates and had enlarged spleen and lymph nodes but smaller than normal thymus, suggesting that the expanding lymphocytes are of B cell origin (Figure 2B and Table S1). Flow cytometry (FC) analysis on blood from mutant animals confirmed that the expanding population contributing to LPD was of the B cell lineage (B-LPD; Figure 2C). These observations suggested that extreme B-LPD could be the cause of death in PSDCKO and N1N2CKO animals.

### RBP-j–Independent (but γ-Secretase–Dependent) Notch Signaling in the Skin Contributes to Longevity

Unexpectedly, *Msx2*-*Cre*/+; *RBP-j^flox^* ^/*flox*^ (RBP-jCKO) mice lived significantly longer (95 days on average) and showed lower WBC counts (∼70,000 cells/μl) compared to mice lacking Notch receptors or γ-secretase (Figure 1B and 1C). Although we could not exclude the possibility that *Cre*-mediated deletion of *RBPSUH* is marginally less efficient than that of *PS1* or *Notch1* and *Notch2*, examination of RBP-j protein in RBP-jCKO animals confirmed its loss in keratinocytes (Figure S1). If RBP-j could actively repress some target genes in the absence of Notch signaling and this repression would be lost upon RBP-j deletion, then the milder RBP-j phenotype could be explained by de-repression of some targets that ameliorated the phenotype [18]. Alternatively, the Notch pathway may have bifurcated; RBP-j-independent (yet γ-secretase-dependent) targets of Notch in skin [10,19] may contribute to the severity of the phenotypes reported here. To distinguish between these possibilities, mice that lack both γ-secretase and RBP-j in the skin were generated (*Msx2-Cre*/+; *PS1^flox^* ^/*flox*^; *PS2* ^–/–^; *RBP-j^flox^* ^/*flox*^; PSDRBP-jCKO). In this genetic experiment, de-repressed targets should suppress the PSDCKO phenotype (i.e., PSDRBP-jCKO would display the RBP-jCKO phenotype [18]). However, if RBP-j-independent targets of Notch contribute to the life span and leukocytosis, then the γ-secretase mutation should be epistatic to RBP-j (i.e., the animal will have the PSDCKO phenotype). A combination of both would be expected to produce an intermediate phenotype.

The PSDRBP-jCKO mice lost RBP-j yet had the same life expectancy as PSDCKO mice (Figure 1B) and had WBC counts comparable to those of PSDCKO (Figure 1C), a result inconsistent with target de-repression. To confirm that these outcomes were Notch-dependent but RBP-j-independent, we also created the triple mutant *Msx2-Cre*/+; *N1^flox^* ^/*flox*^; *N2^flox^* ^/*flox*^; *RBP-j^flox^* ^/*flox*^ (N1N2RBP-jCKO). As with PSDRBP-jCKO animals, the life span of N1N2RBP-jCKO animals was not prolonged by removal of RBP-j (Figure 1B and 1C), indicating that a Notch-dependent activity contributes to the phenotype. This suggested that canonical Notch signaling could not be the sole determinant of the phenotypes described and instead supports the alternative assertion that RBP-j-independent effects of Notch in the skin may contribute to leukocytosis [10,20]. This result provides the first genetic evidence in a vertebrate to support the existence of a bifurcation in Notch signaling downstream of γ-secretase.

### Drastic but Transient Expansion of B-Lymphocytes Results in Severe Systemic Complications in Notch-Deficient Animals

**Figure 3:**
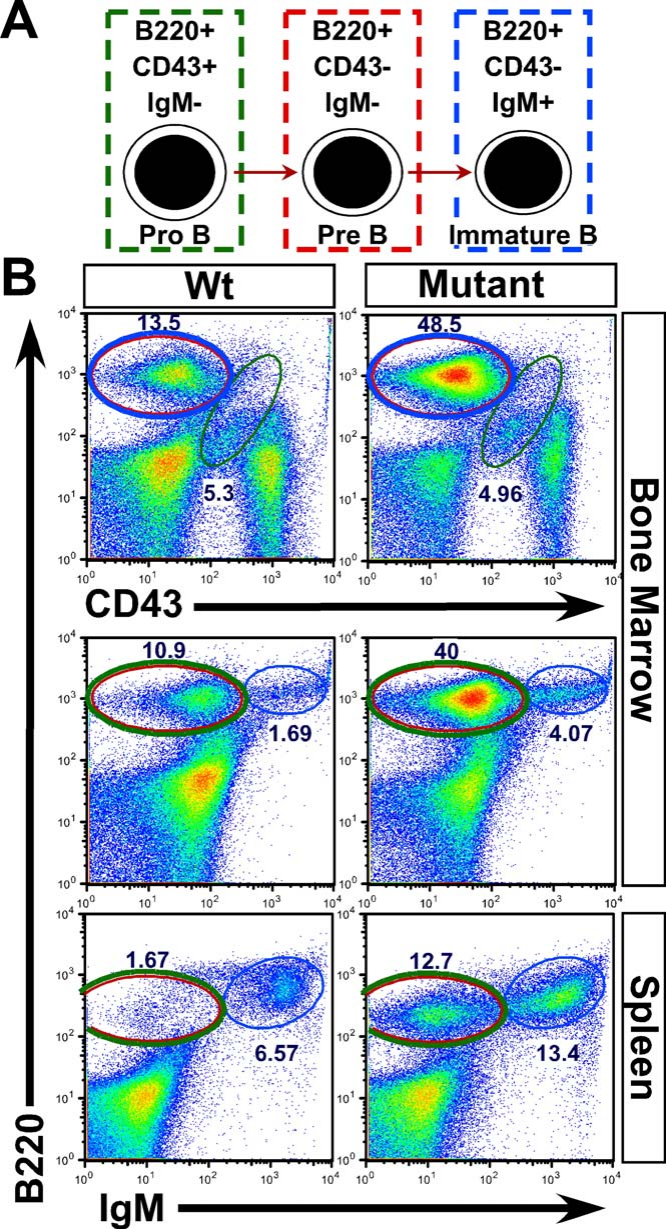
Expanding Pre- and Immature B Cells Cause B-LPD in the Mutant Animals (A) Schematic representation of how surface markers applied (B220, CD43, and IgM) identify pro-, pre-, and immature B cells. (B) FC analyses of BM and spleen cells demonstrate a clear expansion of pre- (red circle) and immature (blue circle) B cells in both central and peripheral compartments of the mutant animals. More than three mice from each genotype (PSDCKO, N1N2CKO, and wild-type littermates) are analyzed. Representative plots are shown.

To understand B-LPD progression better, the WBC count from mutant animals was measured over their life span (Figure 2D). The WBC counts increased exponentially over the first 2 weeks starting at around P4 but plateaued during the third week of life within the same time window when most mutant animals expired. However, to our surprise, the longest surviving mice showed a trend toward normalization of their WBC counts (Figure 2D). A reduction in peripheral B cell number (and thus normalization of WBC count) was more evident in animals with partial loss of Notch signaling and in the longer living RBP-jCKO mice (unpublished data). Accompanying the surge in peripheral WBC count, B220^+^ B cells expanded the spleen (splenomegaly) and infiltrated several vital organs including lung and liver (Figure 2E, 2F, and Figure S2A). To characterize the identity of the expanding cells in B-LPD further, two additional markers, CD43 and IgM, were applied to segregate pro-, pre-, and immature B cell populations by FC (Figure 3A). The FC analysis showed that pre-B cells (B220^+^CD43^–^IgM^–^) constituted the majority of the expanding population in both BM and periphery (Figure 3B, red ovals). Immature B cells also expanded, as would be expected if differentiation of pre- to immature B cells persisted in the mutant animals (Figure 3B). The ability of pre-B cells to differentiate and the high WBC counts reached at an early age are consistent with a polyclonal origin of this B-LPD [21]. Large expansion of immature B cells was associated with elevated IgM levels, which precipitated at low temperature, a condition known as cryoglobulinemia (Figure S2B). Overall, despite the short duration of B-LPD in Notch-deficient mice, we hypothesized that extremely high WBC count, leukostasis, cryoglobulinemia, anemia, and infiltration of B cells into vital organs conspired with the skin phenotype to produce cachexic mice that failed to thrive during the early stages of postnatal development.

### Blocking B-LPD Prevents Early Lethality in the Mutant Mice

**Figure 4:**
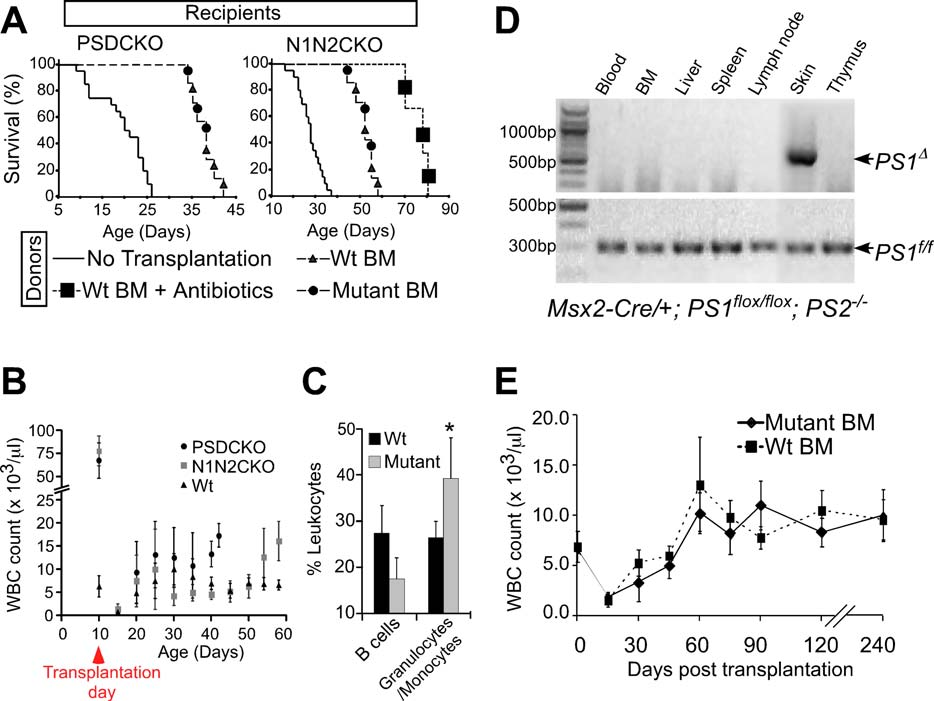
BMT Cures the Lethal B-LPD in the Mutant Animals and Confirms the Local Deletion of Notch Pathway in the Skin as the Sole Driver of B-LPD (A) Both PSDCKO and N1N2CKO mice, lethally irradiated and transplanted with BM from their wild-type (*n* = 4) or mutant (*n* = 3) littermates (at ∼P10), live significantly longer than their untransplanted mutant counterparts (*n* = 20; *p* < 0.001, log rank test). In the N1N2CKO group, three mice that are transplanted with wild-type BM also receive daily antibiotic treatment after BMT, leading to their significantly longer life span compared to other transplanted mutants (*p* < 0.001, log rank test). B-LPD does not recur in the transplanted mutant animals shown by (B) their persistently low WBC count during their post-BMT life (data are drawn from four animals in each genetic group) and (C) on average lower B220^+^ B cell percentage in their blood as compared to the wild-type recipients, measured 25 d after transplantation (four PSDCKO and four wild-type littermates are compared). Note that at this stage the transplanted PSDCKO animals develop granulocytosis with significantly higher granulocyte/monocyte percentage (*, *p* < 0.05). (D) *Msx2*-*Cre*-mediated *PS1* gene deletion (*PS1* ^Δ^) is only detectable in the skin of the PSDCKO animals. The data are representative of three independent tests. DNA samples are prepared from PSDCKO animals with WBC count >100,000 cells/μl. BM samples are unfractionated and include BM stroma. (E) Wild-type mice (∼P10) receiving BMT from their mutant (*n* = 12, both N1N2CKO and PSDCKO are included) or wild-type (*n* = 12) littermates reconstitute normal hematopoiesis and have a normal life span without developing B-LPD.

To determine whether B-LPD was an important contributor to early death, we performed an allogeneic bone marrow transplantation (BMT) experiment with mutant animals as the recipients. Lethally irradiated mutant mice transplanted with BM derived from their wild-type littermates around postnatal day 10 (P10) lived significantly longer than their untransplanted counterparts (Figure 4A). However, the transplanted mutants still died within a few weeks after transplantation, this time because of severe skin phenotypes including exfoliation, bleeding, inflammation, and infection (Figure S2C). When treated with systemic antibiotics, the life span of transplanted N1N2CKO animals was extended further and became comparable to that of RBP-jCKO mice (Figures 4A and 1B). However, antibiotic treatment of PSDCKO mice did not extend further their life span due to the greater severity of skin disease in these animals (Figure S2C). The WBC counts and FC analyses showed no reoccurrence of B-LPD in the transplanted mutants (Figure 4B and 4C). However, a significant expansion of granulocytes/monocytes was observed in the peripheral blood of transplanted mutant animals during the final days of life with WBC counts reaching ∼20,000 cells/μl (Figure 4B, 4C, and Figure S3). Similar granulocytosis accompanied by elevated peripheral T-lymphocyte percentage was observed in RBP-jCKO mice of the same age (Figure S3).

The observations detailed above suggested that if B-LPD was controlled, then life expectancy of the mutant animals could be increased significantly. Indeed, either a sublethal dose of total body irradiation (∼450 cGy) or focal irradiation of the mutant animals extended their life span. In both cases, life expansion correlated with a delay in B-LPD surge (Figure S4). Taken together, these experiments strongly demonstrated that B-LPD, acquired by animals with Notch- or γ-secretase-deficient skin, was a critical mediator of early lethality. However, the short life expectancy of transplanted mutants and RBP-jCKO was related to their skin disease, infection, and granulocyte/monocyte expansion, which again occurred in a Notch dose-dependent manner and in all adult animals lacking canonical Notch signaling (unpublished data and Dr. Freddy Radtke, personal communication).

### B-LPD Is a Non-Autonomous Consequence of Losing Notch or γ-Secretase in Skin Keratinocytes

Considering the importance of Notch signaling at various stages of lymphopoiesis [22,23] and the potential for ectopic expression of *Msx2-Cre* in a hematopoietic organ, we asked whether deletion of Notch in BM or any hematopoietic organ might have caused B-LPD. To map the sites of *Msx2-Cre* activity thoroughly, we applied the following approaches.

First, using a PCR protocol designed to detect the deleted allele of *PS1* (*PS1* ^Δ^), we confirmed that neither hematopoietic cells nor any hematopoiesis-related organ experienced *Cre*-mediated deletion of *PS1* in PSDCKO mice (Figure 4D). Of note, we collected peripheral blood from the mutant animals at peak WBC counts, which thus was composed mostly (>90%) of expanding B cells, but still found no evidence of PS1 deletion. To rule out the possibility that loss of Notch signaling in a small subset of BM stromal cells could drive B cell expansion in the mutant animals, we re-analyzed *Prx1*-*Cre*; *PS1^flox^* ^/*flox*^; *PS2* ^–/–^ mice in which *Cre* is active in BM stroma, the osteoblasts [24,25], and the osteoclasts [26]. The WBC analysis in all these mice showed no sign of B cell expansion (unpublished data), indicating that another organ provided the trigger for B-LPD. Next, we analyzed two reporter lines, *Msx2*-*Cre*; *ZEG*/+ and *Msx2*-*Cre*; *Rosa26R*/+. Only the skin was marked by these reporters (unpublished data) with no detectable reporter staining in any hematopoietic lineage or organ. Together, these findings led us to hypothesize that B-LPD was driven non-autonomously in wild-type B cells as a consequence of reduced Notch signaling in the skin.

To test this hypothesis, we applied the allogeneic BMT paradigm to ask whether hematopoietic stem cells isolated from the mutant animals could propagate B-LPD in normal recipients. The BM derived from mutant or wild-type littermates was equally competent in its ability to engraft in lethally irradiated wild-type littermate hosts and reconstitute a complete hematopoietic system that sustained a normal WBC count in the recipient animals over several months of follow-up (Figure 4E). The PCR analysis confirmed the complete repopulation of the recipients' hematopoietic system by donor-derived BM (Figure S5B). In addition, BM transplanted from mutant or wild-type animals into sublethally irradiated nonobese diabetic/severe combined immunodeficiency (NOD/SCID) mice were indistinguishable in their ability to rescue the recipients (Figure S6). Collectively, these findings confirmed the non-autonomous nature of B-LPD and implicated the skin as the primary organ responsible for the disease in Notch/γ-secretase-deficient animals.

### High Levels of TSLP Strongly Correlate with the Intensity and Timing of B-LPD in Notch-Deficient Animals

**Figure 5:**
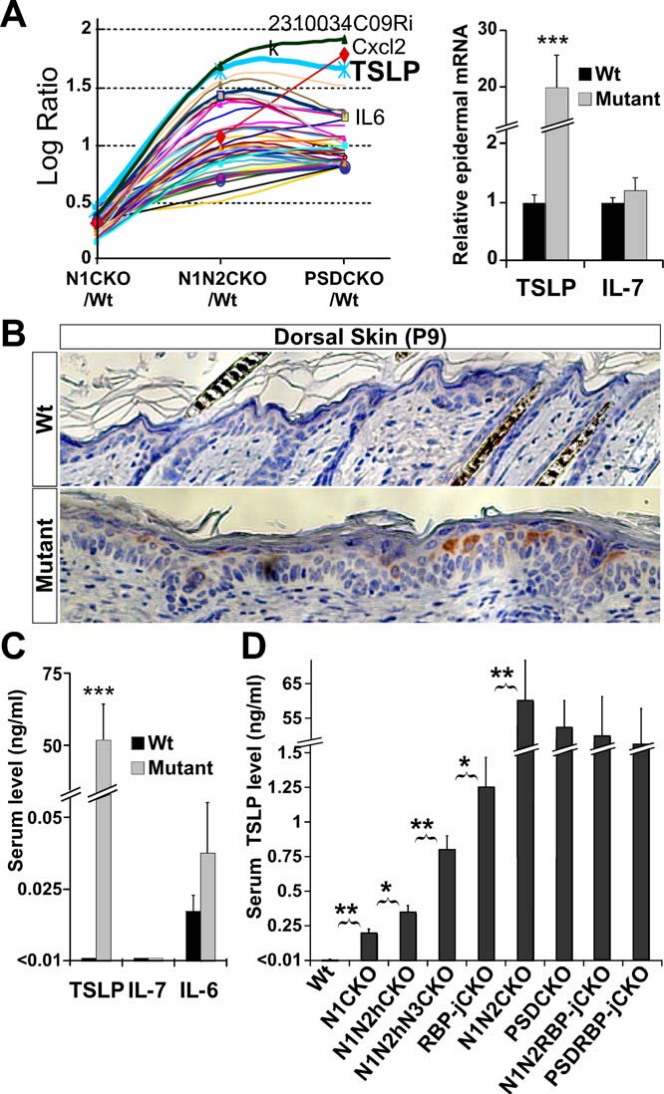
TSLP Is Highly Expressed in the Skin Lacking Total Notch Signaling, Leading to Its High Systemic Levels Such elevated serum levels inversely correlate with Notch dosage in the skin. (A) Modified trend analysis performed on microarray data from P9 mutant and wild-type total-skin RNA samples identifies *TSLP* as the most up-regulated cytokine gene in the mutants. The epidermal overexpression of *TSLP* in the mutant mice is confirmed by qRT-PCR. (B) TSLP is mainly produced by suprabasal keratinocytes in the mutant epidermis (200× magnification). (C) Unlike IL-6 and IL-7, TSLP serum protein levels measured on ELISA are highly elevated in the mutant animals. (D) Serum analyses during the second week of life show that TSLP levels increase as more alleles of Notch are removed from the skin. Data are extracted from comparing three mutant (of each genotype) and three wild-type littermates (*, *p* < 0.05, **, *p* < 0.01, ***, *p* < 0.0001).

Having identified skin keratinocytes as the only cells in which *Cre*-mediated deletion of Notch signaling was occurring, we hypothesized the existence of (an) epidermally derived cytokine(s) capable of driving B cell expansion that accumulated to high systemic levels in inverse correlation with Notch dose. To search for such factor(s), we performed microarray analysis of mutant and wild-type total-skin RNA samples collected at P9. Given the dose–response observed with life expectancy and B-LPD, we performed a modified trend analysis asking for transcripts that were modestly elevated in Notch1-deficient (N1CKO) skin but substantially elevated in N1N2CKO or PSDCKO skin. A small subset of altered transcripts, highly enriched for chemokines and chemoattractants, displayed the desired trend (Figures 5A and S7 and Table S2). Among them, *TSLP* was the second most abundant transcript and the only epidermally derived cytokine capable of driving fetal B cell proliferation in mouse [17,27].

Quantitative reverse transcription PCR (qRT-PCR) on epidermal mRNA samples confirmed a ∼20-fold increase of *TSLP* mRNA in mutants (Figure 5A and Table S2), and immunohistochemical analysis on skin sections identified suprabasal keratinocytes as the source of TSLP (Figure 5B and Figure S8). ELISA measurements detected TSLP levels >5000-fold above the normal levels in sera from mutant mice (∼50 ng/ml versus <10 pg/ml) that were already detectable at birth (unpublished data). A comprehensive serum analysis failed to detect differences in any other cytokine or autoimmune signature that could provide an alternative mechanism for B-LPD in the mutant mice (Table S3), including IL-7, the main cytokine implicated in B cell development. Interleukin 6 (IL-6), the only other cytokine implicated in B-lymphopoiesis, which showed moderate yet significant up-regulation in the skin microarray trend analysis, was elevated <2-fold in serum (Figure 5C). Examination of the sera from entire allelic series of Notch-deficient mice by ELISA confirmed and extended our trend analysis: The TSLP levels showed a strong inverse correlation with the dose of Notch signaling and life expectancy (Figure 5D) and a direct correlation with WBC counts of the mutant animals (Figures 1B, 1C, 5D, and Figure S9).

### Increasing TSLP Levels Is Sufficient To Cause B-LPD in Mice but Only during the First Weeks of Life

**Figure 6:**
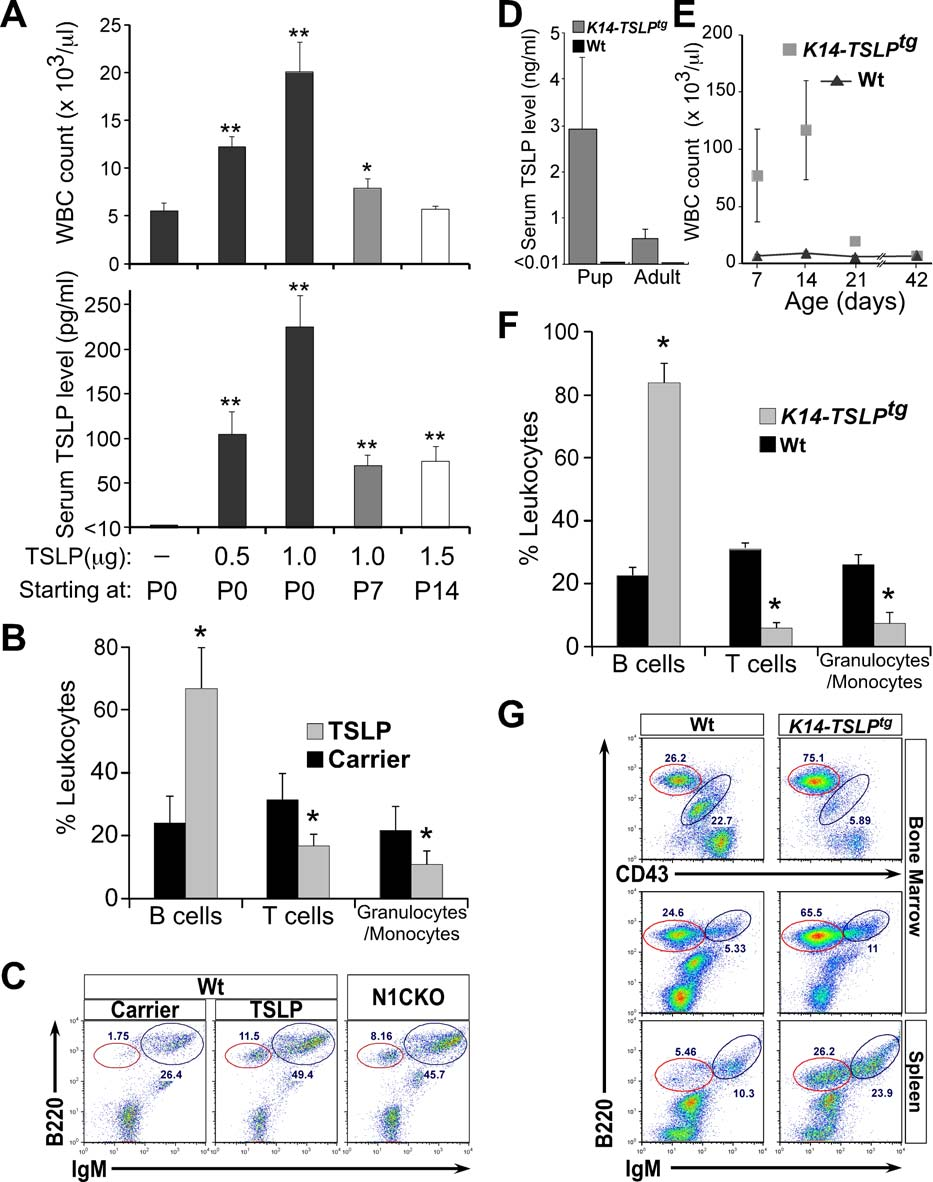
TSLP Is Sufficient To Drive B-LPD in Newborn Animals (A) Wild-type mice injected intravascularly with carrier alone (PBS), 0.5, 1, or 1.5 μg of recombinant mouse TSLP daily for 7 days show a dose-dependent increase in serum TSLP levels and WBC counts (measured 12 h after the last dosing) only if the treatment starts at P0. The impact of elevated serum TSLP on WBC count is diminished or absent when the treatment starts on P7 or P14, respectively (*n* = 3, for each condition; *, *p* < 0.05 and **, *p* < 0.01). (B and C) FC analysis on peripheral blood from mice receiving 1 μg of TSLP daily for 7 days starting at P0 confirms that the increase in WBC count is because of the expansion of pre- (red circles) and immature B cells in the periphery, identical to N1CKO newborns that have similar serum TSLP levels and WBC counts (*n* = 3, for each group; *, *p* < 0.05). (D) *K14-TSLP^tg^* mice phenocopy the neonatal B-LPD in RBP-jCKO animals; they have highly elevated serum TSLP levels at several time points during the first two weeks of life (averaged as pup). Adult animals (>P21) also have elevated TSLP in their serum but to a lesser degree than in the pups. (E and F) In addition, *K14-TSLP^tg^* mice have high WBC counts due to B-LPD during the first few weeks of life (*, *p* < 0.05). (G) FC analysis identifies the expanding population of cells causing B-LPD as pre- (red circle) and immature B cells. TSLP measurements and FC analyses are performed on three *K14-TSLP^tg^* mice and three wild-type littermates. WBC counts are obtained from six mice in each group.

Collectively, RNA and protein analyses created a consistent picture pointing to TSLP as the likely candidate satisfying most of the criteria for the B-LPD-inducing agent: sensitivity to reduction in Notch dose, systemic availability, and the ability to promote B cell development [17,27]. However, such a proliferative role for TSLP has not been demonstrated previously. To satisfy Koch's postulate for disease causation, we injected recombinant mouse TSLP into wild-type mice. Daily injection of the animals with TSLP starting at birth and continuing for 7 days led to a dose-dependent elevation of WBC count (Figure 6A). The FC analysis on peripheral blood from the mice injected with TSLP identified the expanding cell population as B220^+^ B-lymphocytes, not seen in mice receiving carrier alone (Figure 6B). Further analysis of peripheral blood from wild-type mice receiving 1 μg of TSLP identified the expanding B cell population as pre- and immature B cells (Figure 6C). Injecting wild-type animals with 1 μg of TSLP daily resulted in steady-state serum TSLP levels of ∼250 pg/ml, comparable to that in N1CKO animals (Figures 6A, 5D, and Figure S9). Likewise, WBC counts in these animals were also indistinguishable from those seen in N1CKO mice at P8 (Figures 6A, 1C, and Figure S9). Thus, elevated TSLP levels were sufficient to cause mild B-LPD in an otherwise normal newborn mouse. Significantly and in agreement with published reports, no surge in WBC count was detected when the treatment regimen was initiated at P14 or later (Figure 6A; [28,29]). To demonstrate a correlation between endogenous levels of TSLP and high WBC counts, we analyzed *K14-TSLP^tg^* transgenic mice [30]. This analysis revealed high serum TSLP levels of ∼3 ng/ml in *K14-TSLP^tg^* newborns and, as predicted by our hypothesis, neonatal B-LPD similar to that observed in RBP-j-deficient animals (Figure 6D–G and S9). Importantly, B-LPD in *K14-TSLP^tg^* newborns developed in the absence of any overt skin morphology (unpublished data), indicating that elevated TSLP is not impeding epidermal differentiation. Consistent with the findings above, *K14-TSLP^tg^* animals also developed B-LPD only during the neonatal period, which disappeared later in life despite elevated serum TSLP levels (Figure 6D and 6E). This experiment confirmed that *TSLP* overexpression after the neonatal period did not sustain B-LPD in mice.

To ask if B-LPD represented the confluence of high TSLP with a responding, fetal pre-B cell population, we performed fetal liver transplantation (FLT) into lethally irradiated mutant animals. Surprisingly, FLT reconstituted normal adult hematopoiesis in the recipients, suggesting that fetal pre-B cells in an adult niche microenvironment lost their ability to respond to high levels of TSLP (Figure S10). To ask if continuous exposure to high TSLP levels sustained a responding pre-B cell population, we performed a third allogeneic BMT experiment in which BM from the mutant animals was transplanted into their lethally irradiated mutant littermates (which continued to produce high TSLP levels, unpublished data). Again, the donor-derived BM reconstituted normal adult hematopoiesis in the recipients, curing their B-LPD, consistent with the limited temporal window in which B-LPD can develop (Figure 4A). These results confirmed that B-LPD developed as a result of exposure to high TSLP levels in the perinatal period.

### TSLP Overproduction Is a General Response to Structural Abnormalities in Notch-Deficient Skin before Birth

**Figure 7:**
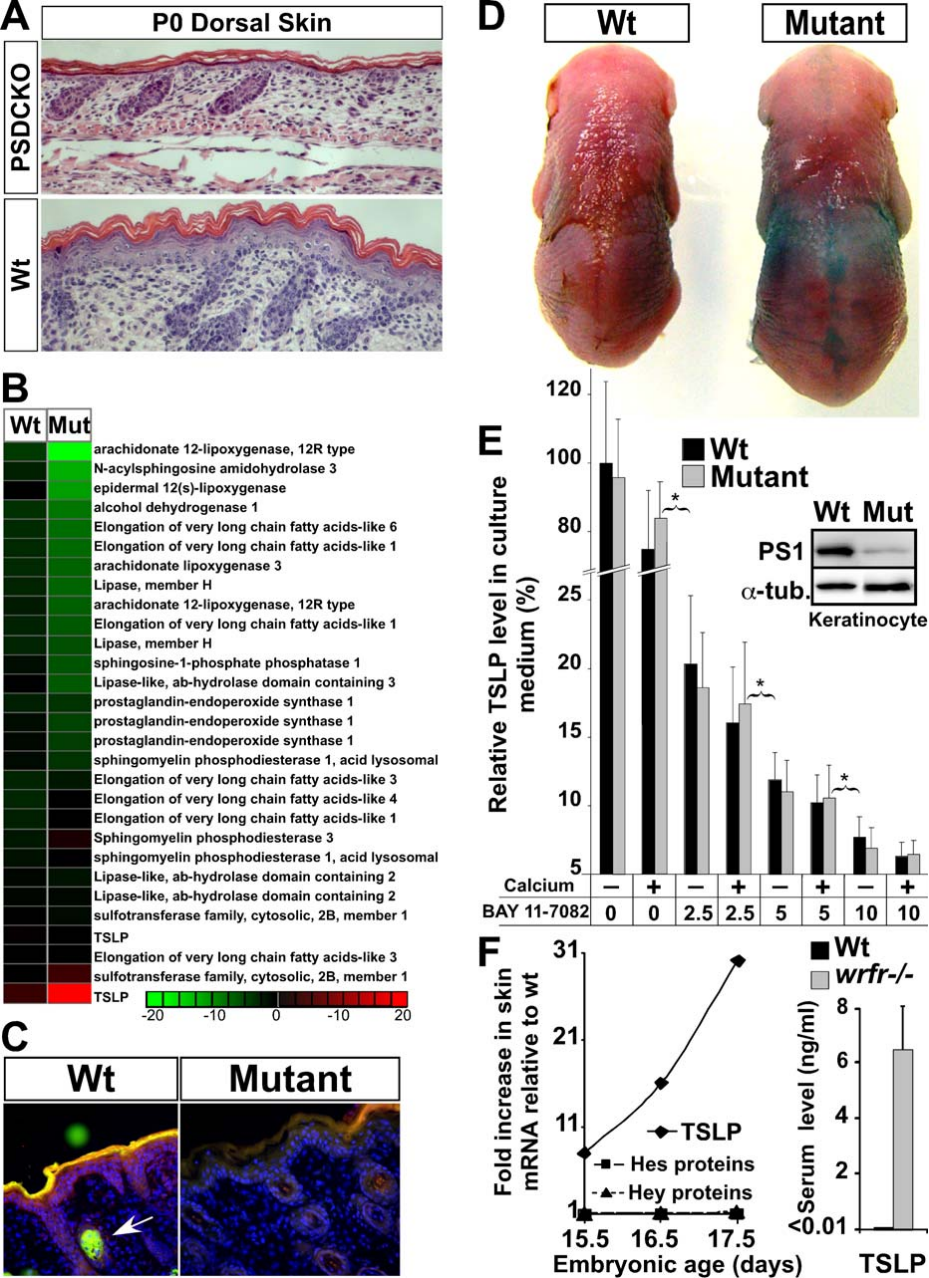
Skin-Barrier Defect Explains How Loss of Notch Signaling in the Skin Leads to Elevated TSLP Production (A) P0 PSDCKO dorsal skin shows a severe epidermal differentiation defect (loss of spinous and granular layers) compared to wild-type littermate (200× magnification). (B) Heat map of microarray data on P9 epidermal RNA shows down-regulation (green) of genes involved in epidermal lipid biogenesis in the mutants versus their wild-type littermates. Note that *TSLP* probes are included as a control. (C) The Nile Red florescent staining of the polar (red) and non-polar (green) lipids in the epidermis of a P5 PSDCKO mouse demonstrates a deficient lipid coat in γ-secretase-deficient (mutant) versus unaffected areas (wild type) of the epidermis [48]. Note the prominent green florescent staining of the sebaceous gland in the wild-type section (arrow), which is absent in the skin lacking Notch signaling [14]. (D) The dye penetration assay shows a defect in the skin barrier of the mutant animal at E18.5. (E) Keratinocytes cultured from the mutant (PSDCKO) or wild-type littermates in the presence or absence of calcium release similar yet significant amounts of TSLP into their medium. However, keratinocyte TSLP production decreases by inhibiting NFκB signaling (BAY 11–7082: 2.5, 5, 10 μM; *: *p* < 0.01) in a dose-dependent manner (data are accumulated from three independent experiments; TSLP levels are presented relative to that of wild-type cells in the absence of calcium and inhibitor). The immunoblot panel confirms that the mutant keratinocyte are deficient in PS1 protein. (F) The *wrfr* ^–/–^ embryos show a surge in the skin *TSLP* expression around the time of skin-barrier formation, as detected by microarray. Note that known Notch targets, Hes and Hey proteins, are not altered in *wrfr* ^–/–^ mice. *TSLP* up-regulation translates into its highly elevated serum levels in *wrfr* ^–/–^ mice at birth (*n* = 3; *p* < 0.0001).

Loss of Notch signaling in skin keratinocytes leads to elevated epidermal TSLP production and high serum TSLP levels, reaching a maximum only when all Notch proteins or γ-secretase are removed (Figure 5). The *TSLP* overexpression thus may be a general response of epidermal keratinocytes to differentiation (and skin-barrier) defect [5]. Notch-deficient epidermis was defective in epidermal differentiation, leading to incomplete formation of upper spinous and granular layers, as reported in RBP-j-deficient animals (Figure 7A; [6]). This was accompanied by global down-regulation of skin lipid biosynthetic enzymes and reduced epidermal lipid content (Figure 7B and 7C and Table S4). Consequently, a defect in skin-barrier function was detectable directly by using the dye exclusion assay; this procedure stains areas with defective barrier, and indeed, only the stereotypical pattern of *Cre*-expression was stained in the mutant animals, consistent with a barrier defect in γ-secretase-deficient keratinocytes (Figure 7D; [6]).

To further ascertain if *TSLP* overexpression is a consequence of Notch loss or a consequence of defective differentiation/barrier formation, we isolated keratinocytes from mutant and wild-type pups and measured TSLP in the culture medium of cells grown on plastic. Both mutant and wild-type keratinocytes fail to form a fully differentiated epidermis under these conditions, and both secreted similar and significant levels of TSLP, as would be expected if TSLP overproduction was a general consequence of abnormal keratinocyte differentiation and not a specific consequence for loss of Notch signaling (Figure 7E). Because NFκB signaling, a potent inducer of differentiation, can activate TSLP [31,32], we asked if this pathway was responsible for *TSLP* expression in cultured keratinocytes. Addition of an inhibitor of NFκB signaling, BAY 11–7082 [33], abrogated TSLP production in a dose-dependent manner in both wild-type and γ-secretase-deficient keratinocytes (Figure 7E). To solidify the conclusion that TSLP overproduction is a readout of failed differentiation and not a specific repressed target of Notch signaling, we analyzed *wrfr* ^–/–^ mice that lack fatty acid transport protein 4 (FATP4) and die at birth because of severe epidermal differentiation and skin-barrier defects [34]. As in Notch-deficient mice, B-LPD was not yet detectable in *wrfr* ^–/–^ mice at birth. These mice, however, did show a substantial surge in skin *TSLP* transcript levels around the time of stratification and barrier formation (E15.5–17.5), which led to elevated serum TSLP levels at birth (Figure 7F). Because *FATP4* expression is not altered in Notch-deficient skin (unpublished data) and Notch pathway targets are not altered in *wrfr* ^–/–^ skin (Figure 7F), this observation provided an independent confirmation that up-regulation of *TSLP* expression is a common readout of differentiation/barrier formation defects and not a repressed target of Notch signaling.

## Discussion

### Skin-Specific Deletion of Notch Signaling Leads to a Lethal B-LPD

**Figure 8:**
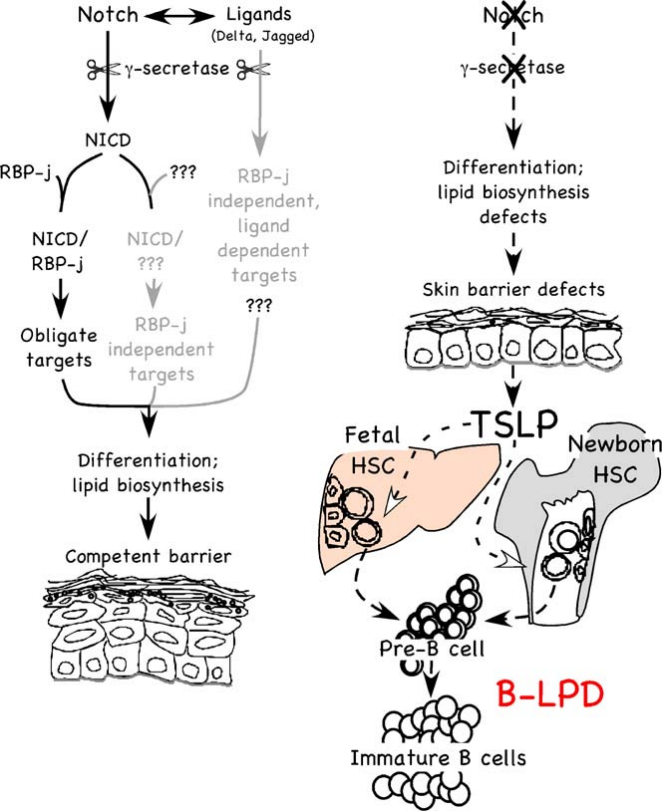
Model of the Role of the Notch Signaling Pathway in Setting up Competent Skin Barrier and Preventing B-LPD in Newborn Animals RBP-j-dependent and independent Notch signaling is required for proper epidermal differentiation and lipid biogenesis in the skin to form a competent barrier. The RBP-j-independent signals may be non-cell autonomous. Embryonic loss of Notch signaling leads to aberrant epidermal differentiation/lipid biogenesis and, therefore, the defective barrier formation. In response, TSLP is released by keratinocytes that fail to differentiate, triggering a systemic B-LPD in newborns that results in neonatal lethality at the highest level.

This report details the mechanism by which a local perturbation to the skin induced a lethal systemic disease. We demonstrate that progressive loss of Notch signaling in the embryonic ectoderm caused a dose-dependent impairment of epidermal differentiation and reduced lipid biogenesis, stimulating keratinocytes to secrete excess TSLP into systemic circulation. The pathophysiological consequence of persistent differentiation/barrier defects and the subsequent accumulation of TSLP had a potent proliferative effect on fetal/newborn B-lymphopoiesis, causing an exceptional expansion of pre- and immature B cell populations in newborn animals (i.e., neonatal B-LPD; Figure 8). A severe B-LPD with its systemic complications including infiltration of B cells into various vital organs, anemia, and cryoglobulinemia explained the early death of the animals lacking total (canonical and noncanonical) Notch signaling in the skin. Indeed, we were able to extend the life span of the mutant newborn animals simply by suppressing their B-LPD through either BMT or sublethal or focal irradiation administered before the onset of full-blown B-LPD in the second week of life. The ameliorating effects of these treatments also reflect the fact that pre-B cells emerging in the adult BM niche are refractory to high TSLP levels (see below).

### Novel Function of TSLP Explains the Main Features of B-LPD in Notch-Deficient Newborns

A growing body of work has provided extensive evidence that *TSLP* overexpression in skin or lung epithelia can cause local allergic inflammation and subsequent development of atopic dermatitis and asthma, respectively [16,35,36]. However, the systemic consequences of *TSLP* overexpression were not clear [29,37]. In this report, we describe the mice in which defects in skin differentiation drive endogenous *TSLP* expression, beginning before birth. We find that TSLP levels are correlated directly with the degree of disruption in differentiation. This enabled us to recognize a novel consequence for TSLP elevation unique to fetal/newborn hematopoiesis and to ascribe TSLP production to failure in skin differentiation rather than its cause, as high levels of TSLP did not alter skin differentiation in newborn *K14-TSLP^tg^* mice.

It has been recognized that TSLP has a differential effect on fetal versus adult B-lymphopoiesis [27,38], despite the presence of fully functional TSLP receptors on both fetal and adult B cells [27,39]. In addition, in vitro results show that a liver-derived fetal pre-B cell line, NAG8/7, but not a BM-derived adult pre-B cell line, IxN/2B, proliferate in response to TSLP [40]. Consistent with these findings, we demonstrate that endogenously overexpressed or exogenously supplied TSLP, delivered during the perinatal period, lead to B-LPD and the appearance of a substantial number of pre-B cells (B220^+^CD43^–^IgM^–^) in the periphery in a dose-dependent manner (Figure S9). Transition from fetal/newborn to adult hematopoiesis occurs during the second week of life in the mouse BM [41]. We find that persistently high levels of TSLP do not produce B-LPD after this transition, explaining why B-LPD has not been detected in previous studies [29,37]. The narrow window in which TSLP can drive pre-B cell expansion explains why B-LPD in Notch-deficient and *K14-TSLP^tg^* animals is (1) confined to the first few weeks of life, (2) disappears in animals that experience a less severe disease, and (3) does not recur in the mutant mice after BMT, because adult-type pre-B cells that are reconstituted from the donor BM do not proliferate in response to high TSLP levels [38]. In addition, the fact that B-LPD does not recur in the mutant mice after FLT is consistent with the hypothesis that the niche is instrumental in regulating the response of pre-B cells to TSLP. This is reminiscent of the critical role the hematopoietic stem cell niche plays in defining the fetal versus adult characteristics of hematopoietic stem cells [42].

### Intrinsic Skin Phenotype and Infection Cause Death in Adult Mice Lacking Canonical Notch Signaling

N1N2CKO or PSDCKO mutant animals transplanted with either wild-type or mutant BM do not enjoy a normal life span, dying before 3 months of age. Mice lacking some Notch alleles or lacking RBP-j do not develop lethal B-LPD but instead die prematurely from compromised skin integrity, exfoliation, inflammation, and systemic infection (unpublished data). Although antibiotic treatment delays the death of the mutant animals by controlling their infection, we find persistent granulocytosis even in antibiotic-treated mice, suggesting that systemic infection and the resulting inflammatory response are severe and incurable. Infection is the most likely cause of granulocytosis in the mutant animals; however, it is intriguing to speculate that persistently high levels of TSLP also contribute to this blood disease ([37] and Dr. Freddy Radtke, personal communication).

### *TSLP* Overexpression in Notch-Deficient Skin Is a Common Keratinocyte Response to Skin-Barrier Formation Defect

Notably, we observe a tight correlation between systemic TSLP levels, WBC counts, and degree of Notch loss in the neonatal skin. All Notch paralogs contribute to skin homeostasis, and stepwise reduction of Notch dosage leads to progressive skin perturbation resembling atopic dermatitis (unpublished data). However, we find no evidence arguing for a specific molecular connection between Notch loss and TSLP. Instead, the tight reverse correlation between serum TSLP levels and Notch dosage in the skin highlights the fact that TSLP levels reflect, with great precision, the magnitude of the differentiation defects. Thus, TSLP may be a direct readout of defects in differentiation, a systemic signal that many insults, including Notch loss, activate. To confirm this, we demonstrate that wild-type and mutant keratinocytes release significant, but similar, levels of TSLP when placed in culture in the absence of any exogenous stimulus (e.g., tumor necrosis factor α). In addition, *wrfr* ^–/–^ embryos show a substantial increase in epidermal *TSLP* transcripts in utero around the time of barrier formation. Therefore, TSLP is a keratinocyte “quality control” response to defective differentiation/barrier formation and not a secondary product of functional barrier failure after birth. One model for how the TSLP “sensor” works may be through the compensatory activation of NFκB. However, precisely how Notch loss in vivo leads to NFκB activation remains an important unanswered question that falls beyond the scope of this paper.

A striking finding is that RBP-jCKO mice show lower TSLP levels, a sublethal B-LPD, and improved epidermal morphology when compared to mice lacking total Notch signaling (Figure S11). To differentiate between de-repression of canonical targets and loss of noncanonical target expression, we performed a genetic analysis demonstrating unequivocally that RBP-j-independent Notch activity is of significance in skin homeostasis. This points to the importance of RBP-j-independent effects of Notch, including non-cell autonomous effects on ligand (Delta and Jagged) presenting cells, and only when all these arms of Notch signaling are lost, extreme TSLP levels are reached, causing lethal B-LPD (Figure 8). Identification of the relevant noncanonical targets also falls beyond the scope of this paper.

### Conclusion

In this report, we identified canonical and noncanonical Notch signaling as essential regulators of epidermal differentiation/barrier formation, TSLP as a faithful reporter of keratinocyte differentiation/barrier defects, and B-LPD as the pathological consequence of chronic, perinatal TSLP elevation. Our analysis was done in a physiologically relevant setting: chronic skin-barrier formation defect caused by localized reduction in Notch signaling in a temporal and dose-dependent manner. How keratinocytes directly sense the degree of differentiation/barrier defect and translate such a stimulus into TSLP output remains an important, unsolved question. Nonetheless, the demonstration that defective skin differentiation can drive a lethal systemic B-LPD in mice through *TSLP* overexpression and the observation that human B cells also respond to TSLP [29] bring up a therapeutically important possibility that chronic high levels of TSLP may be an initiating factor in loss of B cell tolerance and/or B-lymphocytosis, a leukemia-like disease in humans. More important, this raises the possibility that skin has a central role in driving a wide variety of inflammatory and humoral diseases in which skin complications are also present.

## Materials and Methods

### Generation of mice.

Compound strains of mice were engineered as described [14]. All animals were maintained in mixed genetic backgrounds; however, littermates were compared whenever possible. All mice were kept in the animal facility under Washington University animal care regulations. In studies related to longevity, mice were monitored regularly for sign of cachexia and failure to thrive; care was taken to reduce competition for affected pups by removal of wild-type littermates unnecessary for the study. Severely affected individuals were left with their dams for their entire life span. Morphological details of the cutaneous phenotypes will be published elsewhere. The following cohort of animals was analyzed: *Msx2*-*Cre*/+; *N1^flox^* ^/*flox*^ (N1CKO), *Msx2*-*Cre*/+; *N1^flox^* ^/*flox*^; *N2^flox^* ^/+^ (N1N2hCKO), *Msx2*-*Cre*/+; *N1^flox^* ^/*flox*^; *N2^flox^* ^/+^; *N3* ^–/–^ (N1N2hN3CKO), N1N2CKO, PSDCKO, RBP-jCKO, PSDRBP-jCKO, N1N2RBP-jCKO, *Prx1*-*Cre*/+; *PS1^flox^* ^/*flox*^; *PS2* ^–/–^, *Msx2*-*Cre*/+; *Rosa26R*/+, *Msx2*-*Cre*/+; *ZEG*/+, *CD4*-*Cre*/+; *ZEG*/+, *K14*-*TSLP^tg^*. Wild-type cohorts in this study included *Cre*-negative littermates, *Msx2*-*Cre*/+; *PS1^flox^* ^/+^; *PS2* ^–/–^, *Msx2*-*Cre*/+; *RBP-j^flox^* ^/+^, and *Msx2*-*Cre*/+; *Notch1^flox^* ^/+^.

### Histology and imunohistochemistry.

For hematoxylin-and-eosin staining, tissue samples were fixed in 4% paraformaldehyde in phosphate-buffered saline (PBS), dehydrated in ethanol, and paraffin-embedded. Tissues blocks were sectioned at 5 μm; Lac-Z staining on E15.5 embryo was performed as previously described [14]. Immunostaining on paraffin-embedded tissue samples was performed with the following biotinylated antibodies: anti-B220 (clone RA3-6B2, BD Pharmingen), anti-F4/80 (Abcam), anti-CD3e (clone 145–2C11, BD Pharmingen), and anti-TSLP (R&D Systems). Horseradish-peroxidase-conjugated streptavidin and DAB substrate kit (Pierce) were used to visualize the signal. For RBP-j staining, anti-RBP-j antibody (clone T6709, Institute of Immunology) was used together with biotinylated anti-rat secondary antibody. Sections were counterstained with hematoxylin.

### Serology.

Serum TSLP, IL-6, and IL-7 levels were measured according to the manufacturer's instructions in the Quantikine mouse TSLP, IL-6, and IL-7 ELISA kits (R&D Systems).

### Antibodies and flow cytometry.

Single cell suspensions from peripheral blood, BM, and spleen were prepared for FC analysis as described [43]. The following antibodies were used: anti-B220 (RA3-6B2) conjugated to fluorescein (FITC), phycoerythrin (PE), or peridinin chlorophyll-a protein-cyanin 5.5 (PerCP-Cy5.5), anti-CD45 (30-F11) conjugated to PerCP-Cy5.5, anti-Ly-6G (1A8), anti-TER-119, anti-Thy-1 (5E10), anti-CD3e (500A2), and anti-CD43 (S7) conjugated to PE, anti-IgM (R6–60.2) conjugated to FITC and PE (all from BD Pharmingen). Stained cells were studied using a BD FACScan Flowcytometer (Cytek Development), and the data were analyzed using FlowJo software.

### Bone marrow transplantation.

For allogeneic BMT, the immunocompetent recipient mice were lethally irradiated with 950 cGy at ∼P10 and transplanted with freshly harvested, unfractionated BM cells from their littermates as previously described [43]. However, NOD/SCID mice received BMT after a sublethal dose of irradiation (300 cGy). In each case, 2 × 10^6^ cells in 100 μl of PBS + 2% fetal bovine serum were injected into the retro-orbital sinus of the irradiated recipient animal. A cohort of transplanted N1N2CKO mice were treated orally with 50 μl of 200 mg/ml cephalexin (Ranbaxy Pharmaceuticaks) twice daily.

### Disease monitoring.

To study disease progression and monitor disease occurrence/recurrence, all mutant and irradiated/transplanted mice were monitored closely over their life spans for signs of weakness, weight loss, and morbidity. Blood samples were collected from the mandibular vein. Hematological analysis (Hemavet 950 analyzer, Drew Scientific) comprised complete blood count including WBCs, platelets, red blood cells, white cell differential counts, and hemoglobin measurements [44]. White cell differential counts were confirmed on blood smears. In addition, blood samples were collected for FC analysis. Moribund mice were euthanized, and peripheral blood, BM, lymph nodes, thymus, lung, liver, and spleen were collected for a comprehensive pathological analysis as described previously [45].

### TSLP injection.

Wild-type mice were injected intravascularly with carrier alone (50 μl PBS) or with carrier containing 0.5, 1, or 1.5 μg of recombinant mouse TSLP (R&D Systems) daily for 7 d starting on P0, P7, or P14. Mice were euthanized 12 h after the last injection, and tissues were collected for analysis. An ELISA assay of serum measured the systemic TSLP level. Complete blood count and FC analyses on BM, blood, and spleen were performed to check for any sign of B-LPD.

### Barrier function assay.

To test barrier function defect, a dye penetration assay was performed as previously outlined [46]. Briefly, intact E18.5 embryos were stained in X-gal (pH 4.5) for 12 h at 37 °C. After X-gal staining and three rounds of PBS wash, the embryos were photographed with a digital camera.

### Keratinocyte culture.

Primary keratinocytes were isolated from the dorsal midline skin of newborn mutant and wild-type littermates. The cells were maintained in 60-mm^2^ dishes with a medium of low calcium concentration as previously described [47]. Keratinocytes were plated at 40% confluence and allowed to double. Confluent plates (80%) were treated with 2.5, 5, or 10 μM NFκB inhibitor (BAY 11–7082) or carrier alone (DMSO). After 24 h, cells were re-fed with inhibitor/DMSO containing fresh medium with or without 2 mM CaCl_2_ to induce differentiation. After 24 h, cells and their media were harvested for analysis.

### Immunoblotting.

Keratinocyte lysates in SDS were immunoblotted to check for PS1 (H-70, Santa Cruz Biotechnology) and α-tubulin (B-5–2, Sigma-Aldrich) as described [15].

### Nile Red stain.

Frozen skin sections, 7 μm, were stained with 0.15 mg/ml Nile Red in 75% glycerol for 2 min and counterstained with DAPI [48].

### PCR and qRT-PCR.

Conventional PCR for *PS1* alleles was done on genomic DNA of most tissues. For blood, fresh or frozen blood samples were used directly as template with KlenTaq10 (DNA Polymerase Technology) supplemented with 1.3 M final concentration of betaine. qRT-PCR was performed as described [15]. The primer sequences are provided in the Text S1.

### Microarray.

Detailed description of microarray analyses on skin or epidermal mRNA samples from P9 Notch-deficient animals and skin mRNA samples from *wrfr* ^–/–^ embryos are provided in the Supporting Information (Text S1).

### Statistical analysis.

The bar graphs present the mean and standard deviation of each measured parameter. Student's *t*-test is applied as the test of significance unless otherwise specified.

## Supporting Information

Figure S1 RBP-jCKO Keratinocytes Lack RBP-j Protein α-RBP-j antibody staining of the P0 dorsal skin from wild-type and RBP-jCKO mice confirms the absence of RBP-j protein in RBP-jCKO keratinocytes at birth (200× magnification). Arrowheads refer to; black, basal layer of the epidermis; green, hair follicle; red, dermal fibroblasts.
(3.5 MB JPG)

Figure S2 Phenotypes of Notch-Deficient Mice before and after BMT (A) B220^+^ B-lymphocytes infiltrate vital organs of P14 mutant animal (200× magnification). Note that B-lymphocytes have filled the bone marrow, interfering with normal hematopoiesis and contributing to the anemia and thrombocytopenia in mutant animals.
(B) Substantial cryoglobulin formation is detected in the mutant serum after incubating the sera at 4 °C for 48 h.
(C) Dorsal and ventral views of BMT-rescued animals show that PSDCKO mice have a faster progressing skin disease than N1N2CKO mice. Due in part to this skin phenotype, BMT-rescued PSDCKO mice have a shorter life span than N1N2CKO mice, reaching the terminal stage at P40.
(3.7 MB JPG)

Figure S3 FC Analysis on Peripheral Blood Samples from N1N2CKO and RBP-jCKO Mice Comparing the profiles obtained at P12 with those seen at P78 demonstrates the transition from B-LPD (P12) to granulocytosis (P78) relative to wild-type controls. B-lymphocyte, T-lymphocyte, and granulocyte/monocyte percentages are shown in blood. Note that to allow the N1N2CKO animal to reach P78 this individual has undergone BMT at P10 and has been receiving daily antibiotic treatment.
(6.9 MB JPG)

Figure S4 Delay in B-LPD Surge Increased Life Span of the Mutant Mice (A) Either sublethal dose of total body irradiation (∼450 cGy) or focal irradiation of liver or thymus significantly extended life span of N1N2CKO and PSDCKO mice (*n* = 3 per genotype for each treatment group; *p* < 0.01, log rank test). Note that N1N2CKO mice live longer than PSDCKO mice, most likely due to a milder skin phenotype.
(B) Monitoring WBC count shows that B-LPD, although delayed, surges in N1N2CKO and PSDCKO mice receiving sublethal or focal irradiation.
(1.1 MB JPG)

Figure S5 Documenting the Success of BMT by Genotyping DNA from peripheral blood of recipient animals analyzed for the presence of *PS1* alleles 25 d after BMT confirms repopulation of the recipients' hematopoietic system by donor-derived BM cells in both (A) rescue and (B) disease propagation experiments. Because there is no *Cre* activity in the hematopoietic system, the difference between homozygous (one band) and heterozygous (two bands) is detected. Controls for the various genotypes are shown in (B).
(2.1 MB JPG)

Figure S6 NOD/SCID Mice That Receive BM from the Mutant Animals Do Not Develop B-LPD (A) NOD/SCID mice receiving BM from mutant or wild-type newborn donors are indistinguishable over 2 mo of follow-up.
(B) The percentage of B220^+^ B cells in mice receiving BM from mutant or wild-type donors is not significantly different.
(C) Genotyping for *PS1* alleles in peripheral blood from NOD/SCID mice 25 days after BMT shows that most of their WBCs are derived from the donor BM.
(3.3 MB JPG)

Figure S7 Trend Analysis of Microarray Data Cytokines/chemokines that are up- or down-regulated in P9 mutant versus wild-type skin mRNA samples are enriched by this analysis. Note the absence of TNFα.
(2.7 MB JPG)

Figure S8 TSLP Immunostaining of P5 Dorsal Skin PSDCKO mice produce TSLP in suprabasal keratinocytes whereas wild-type mice do not (dashed lines demarcate the basement membrane; 200× magnification). For immunoflorescence, Cy-3-conjugated streptavidin is used to detect biotinylated α-TSLP, and sections are counterstained with DAPI nuclear stain.
(729 KB JPG)

Figure S9 Tight Correlation between Serum TSLP Levels and WBC Counts in the Newborn Mice Comparing (A) the serum TSLP levels and (B) WBC counts of all animal cohorts examined in this study (compiled from the data shown in Figure 1, 5, and 6) reveals a tight correlation between the systemic TSLP elevation and WBC count increase in newborn mice. The measurements are obtained from mutant mice in the second week of life and from TSLP-treated wild-type animals at P8 (*n* = 3, for each group; significant difference between adjacent genotypes is highlighted: *, *p* < 0.05, **, *p* < 0.01).
(1.3 MB JPG)

Figure S10 Fetal Liver Transplantation Cures B-LPD in Notch-Deficient Animals (A) Monitoring the WBC counts of the transplanted mice shows no B-LPD recurrence in the mutant animals after FLT (*n* = 3, for each group).
(B) DNA from peripheral blood of transplanted N1N2CKO animals, analyzed for the presence of *Notch1* alleles 25 days after FLT, confirms the repopulation of the recipients' hematopoietic system by donor-derived fetal liver cells.
(C) FC analysis on peripheral blood from transplanted N1N2CKO, RBP-jCKO, and wild-type mice collected 25 days after FLT shows no sign of B cell expansion in the mutant animals.
(D) As with BMT, curing lethal B-LPD in N1N2CKO animals by FLT (*n* = 3) leads to a relative extension in life span compared to untransplanted N1N2CKO mice (*n* = 20; *p* < 0.001, log rank test).
(1.5 MB JPG)

Figure S11 PSDCKO Mice Have a More Severe Skin Phenotype Than RBP-jCKO Counterparts Cross-sections of the P0 dorsal skin from PSDCKO, RBP-jCKO, and wild-type mice show a progressive loss of epidermal differentiation (200× magnification). PSDCKO skin is most severely affected with loss of all epidermal layers other than basal and squamous. A less severe defect is seen in RBP-jCKO skin, correlating with the lower TSLP levels and improved survival of RBP-jCKO compared to PSDCKO animals.
(4.3 MB JPG)

Table S1 Enlargement of the Hematopoietic-Related Organs in the Mutant Animals Measuring the weight of the animals and their organs at P14 reveals that mutant mice are smaller than their wild-type littermates yet have larger spleen and lymph nodes (*n* = 3, for each group). The values are presented as mean ± standard deviation (“a” indicates *p* < 0.001 (compared to wild-type control)).
(35 KB PDF)

Table S2 Data for the Skin Microarray Trend Analysis Shown in Figure S6
Values are shown as log of fold change. Down-regulated genes are labeled with a minus sign.
(44 KB PDF)

Table S3 Serum Analysis of the Mutant Animals Comprehensive (A) antigen and (B) autoimmune analysis on sera collected from P14 mutant (N1N2CKO; *n* = 3) and wild-type (*n* = 3) animals does not identify any other factor besides TSLP that could potentially explain B-LPD in the mutant animals. The antigens with significantly higher levels in mutant serum are marked (values in red). Note that the least detectable dose for each factor is calculated as three standard deviations above the average value obtained measuring 20 blank samples. Therefore, values below the least detectable dose are below accurate detection range. Rules Based Medicine laboratory cutoff values for autoimmune analysis signify the upper limits of normal ratios for each factor measured.
(46 KB PDF)

Table S4 Changes in Selected Group of Lipid Metabolic Enzymes from PSDCKO and Wild-Type Epidermal Microarray For comparison, TSLP probes also are included. The color highlights samples with *p* < 0.005.
(59 KB PDF)

Text S1 Supplemental Materials and Methods (54 KB DOC)

### Accession numbers

Accession numbers for genes mentioned in this paper from the National Center for Biotechnology Information (http://www.ncbi.nlm.nih.gov) are *FATP4* (AJ276492), *NFκB* (AY521463), *Notch1* (NM_008714), *Notch2* (NM_010928), *Notch3* (NM_008716), *PS1* (NM_008943), *PS2* (NM_011183), *RBP-j*, (NM_009035), and *TSLP* (NM_021367).

## Abbreviations

B-LPD: B-lymphoproliferative disorder
BM: bone marrow
BMT: bone marrow transplantation
FC: flow cytometry
FLT: fetal liver transplantation
IL-6: interleukin 6
IL-7: interleukin 7
N: notch
PS: presenilin
qRT-PCR: quantitative reverse transcription PCR
TSLP: thymic stromal lymphopoietin
WBC: white blood cell
