# Correction: Notch-Deficient Skin Induces a Lethal Systemic B-Lymphoproliferative Disorder by Secreting TSLP, a Sentinel for Epidermal Integrity

**DOI:** 10.1371/journal.pbio.0060169

**Published:** 2008-07-29

**Authors:** Shadmehr Demehri, Zhenyi Liu, Jonghyeob Lee, Meei-Hua Lin, Seth D Crosby, Christopher J Roberts, Perry W Grigsby, Jeffrey H Miner, Andrew G Farr, Raphael Kopan

Correction for:

Demehri S, Liu Z, Lee J, Lin MH, Crosby SD, et al. (2008) Notch-deficient skin induces a lethal systemic B-lymphoproliferative disorder by secreting TSLP, a sentinel for epidermal integrity. PLoS Biol 6(5): e123 doi:10.1371/journal.pbio.0060123

Reference 29 was listed incorrectly. The corrected reference is:

29. Astrakhan A, Omori M, Nguyen T, Becker-Herman S, Iseki M, et al. (2007) Local increase in thymic stromal lymphopoietin induces systemic alterations in B cell development. Nat Immunol 8: 522–531.

