# Supplementary figures and images for: Notch-Deficient Skin Induces a Lethal Systemic B-Lymphoproliferative Disorder by Secreting TSLP, a Sentinel for Epidermal Integrity
Source: PLoS Biol. 2008 May 27;6(5):e123. doi: 10.1371/journal.pbio.0060123 (PMC2430908; doi:10.1371/journal.pbio.0060123)

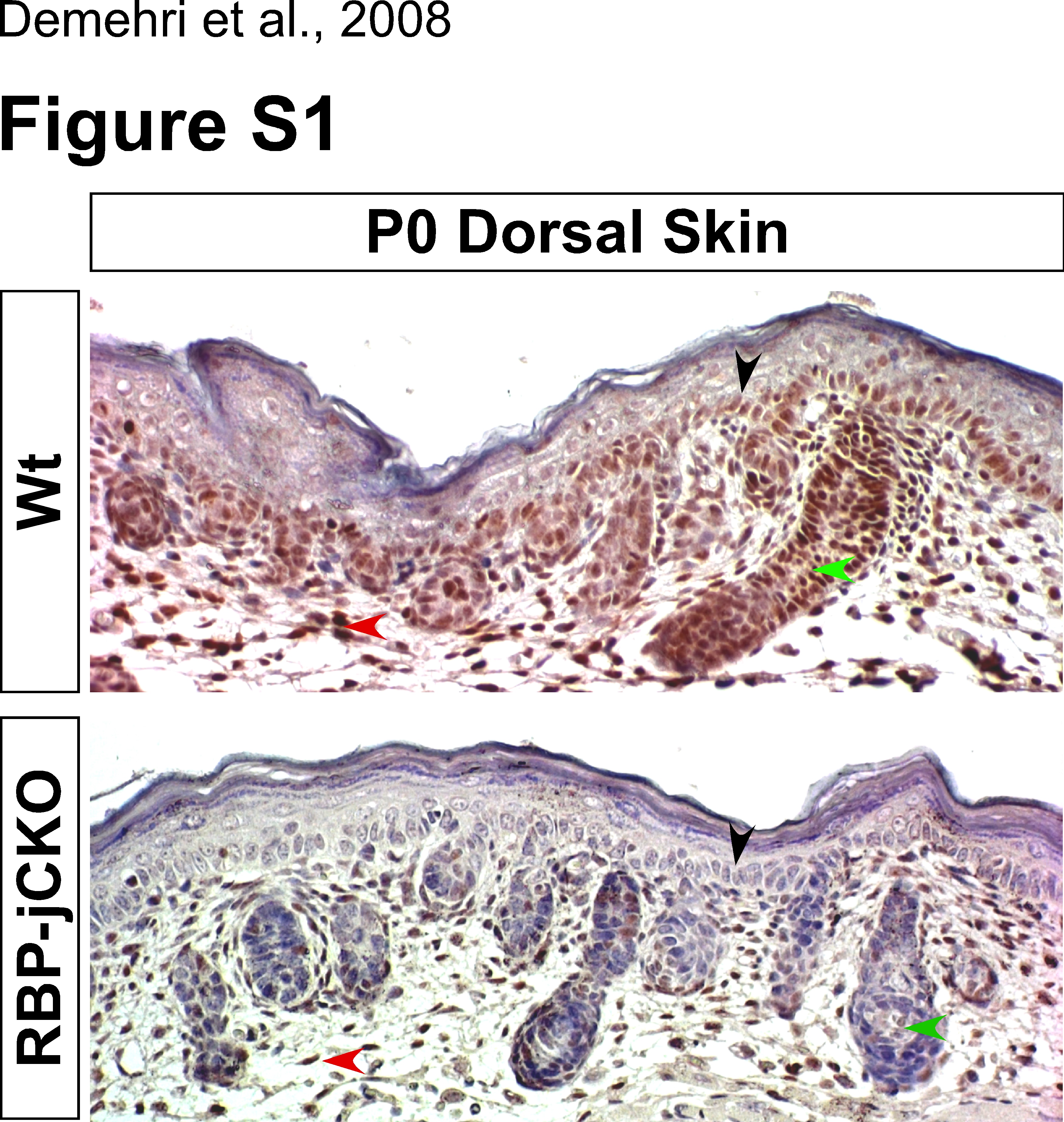

Supplement: Figure S1 — α-RBP-j antibody staining of the P0 dorsal skin from wild-type and RBP-jCKO mice confirms the absence of RBP-j protein in RBP-jCKO keratinocytes at birth (200× magnification). Arrowheads refer to; black, basal layer of the epidermis; green, hair follicle; red, dermal fibroblasts. (3.5 MB JPG) [file pbio.0060123.sg001.jpg]

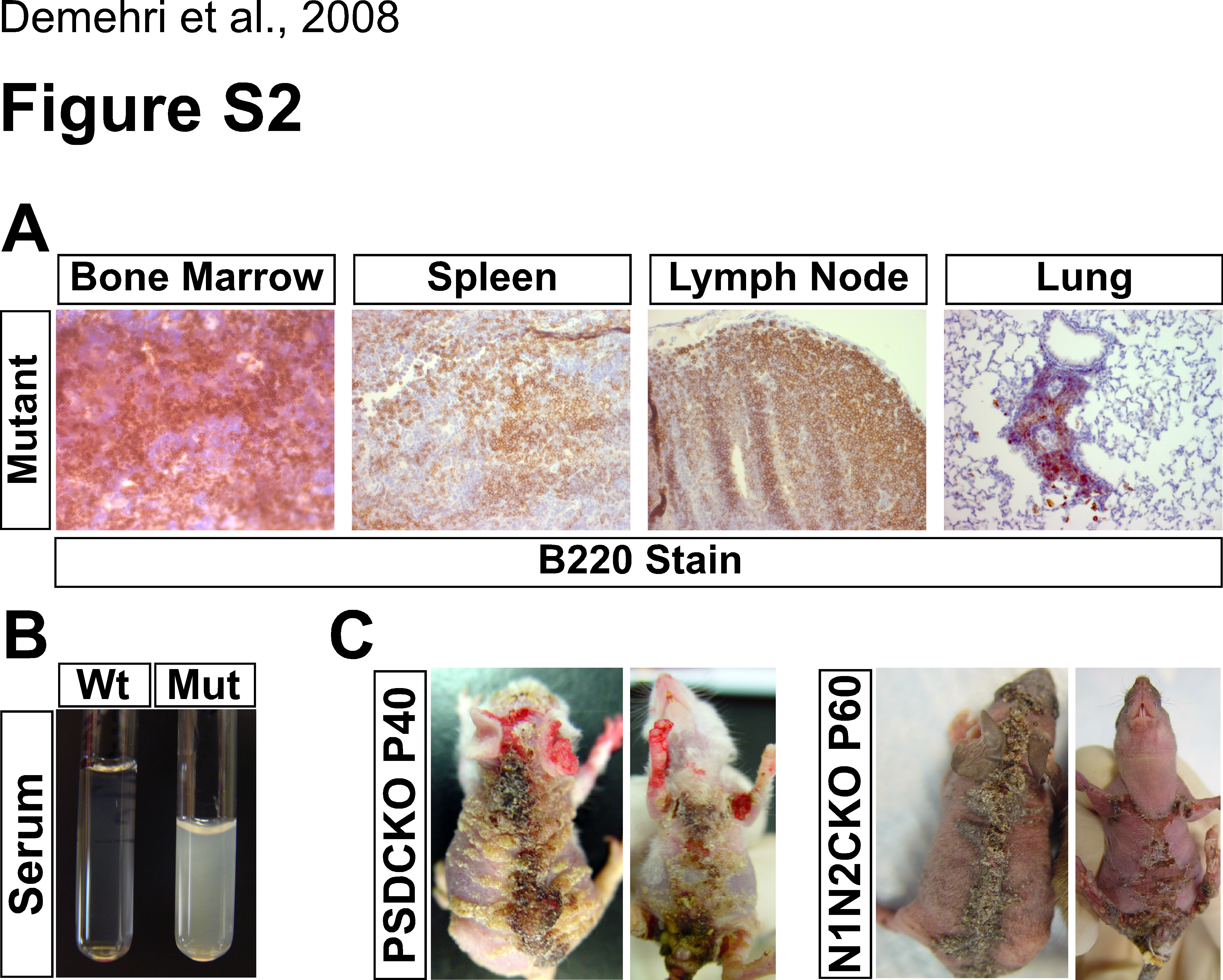

Supplement: Figure S2 — (A) B220+ B-lymphocytes infiltrate vital organs of P14 mutant animal (200× magnification). Note that B-lymphocytes have filled the bone marrow, interfering with normal hematopoiesis and contributing to the anemia and thrombocytopenia in mutant animals. (B) Substantial cryoglobulin formation is detected in the mutant serum after incubating the sera at 4 °C for 48 h. (C) Dorsal and ventral views of BMT-rescued animals show that PSDCKO mice have a faster progressing skin disease than N1N2CKO mice. Due in part to this skin phenotype, BMT-rescued PSDCKO mice have a shorter life span than N1N2CKO mice, reaching the terminal stage at P40. (3.7 MB JPG) [file pbio.0060123.sg002.jpg]

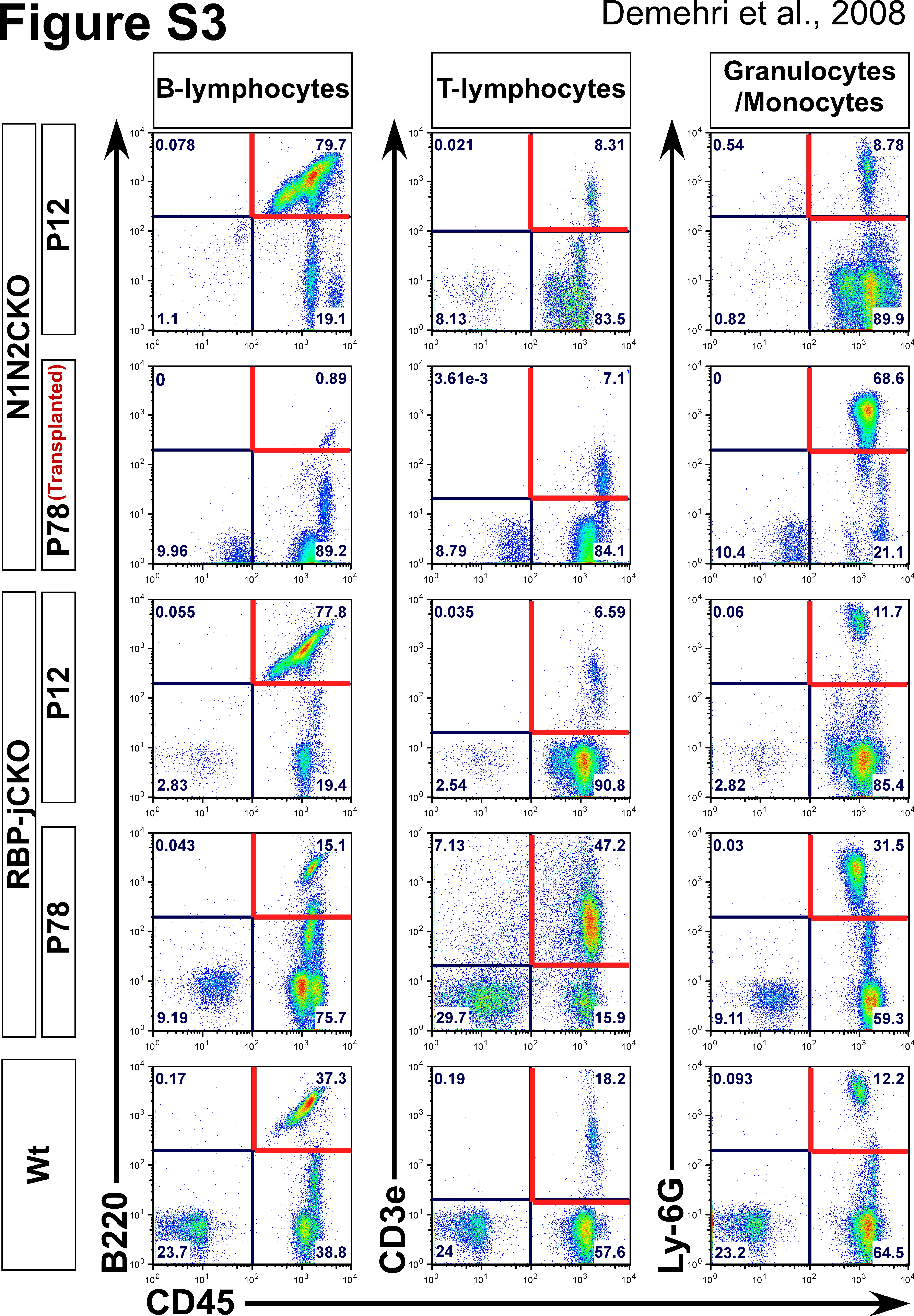

Supplement: Figure S3 — Comparing the profiles obtained at P12 with those seen at P78 demonstrates the transition from B-LPD (P12) to granulocytosis (P78) relative to wild-type controls. B-lymphocyte, T-lymphocyte, and granulocyte/monocyte percentages are shown in blood. Note that to allow the N1N2CKO animal to reach P78 this individual has undergone BMT at P10 and has been receiving daily antibiotic treatment. (6.9 MB JPG) [file pbio.0060123.sg003.jpg]

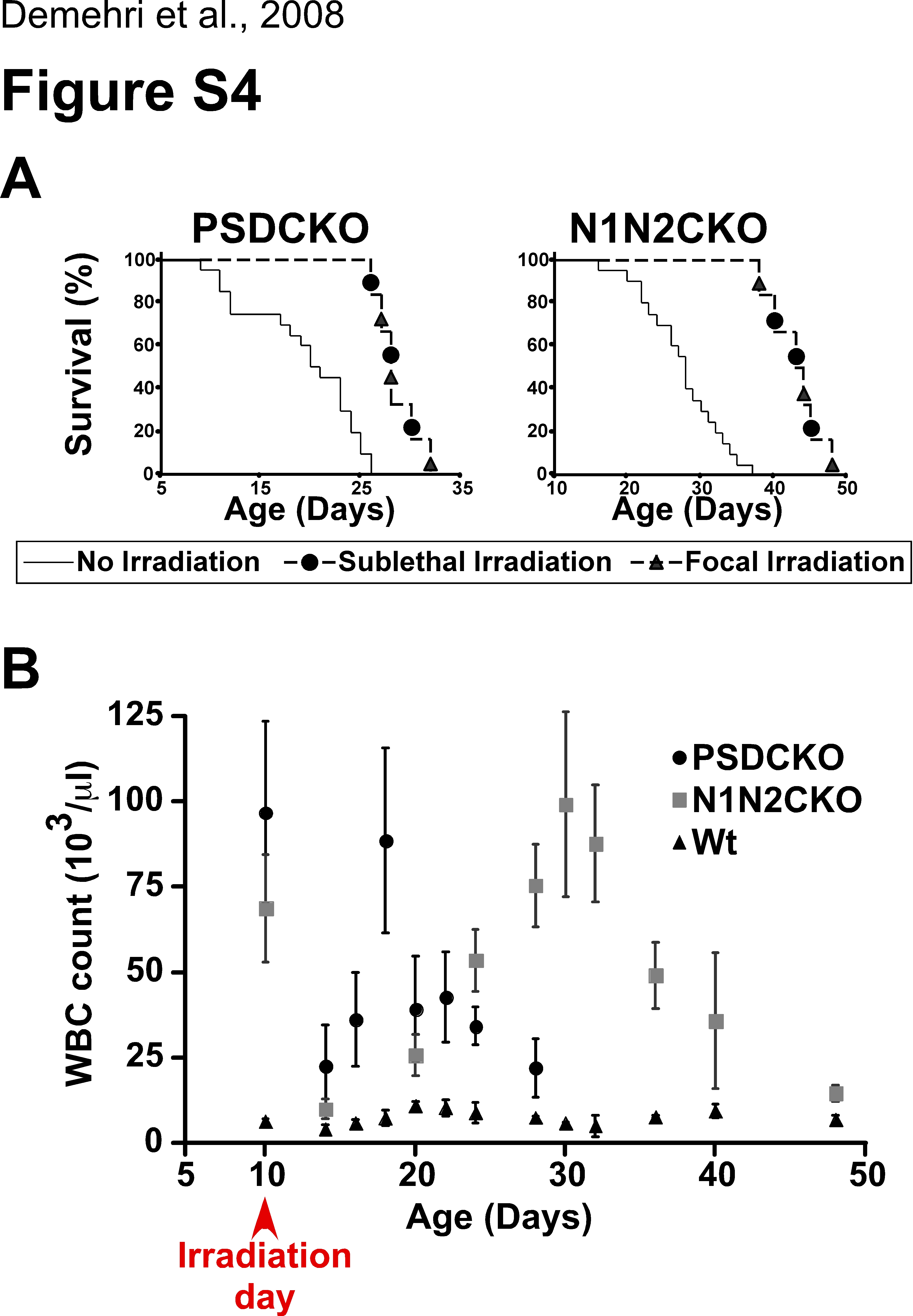

Supplement: Figure S4 — (A) Either sublethal dose of total body irradiation (∼450 cGy) or focal irradiation of liver or thymus significantly extended life span of N1N2CKO and PSDCKO mice (n = 3 per genotype for each treatment group; p < 0.01, log rank test). Note that N1N2CKO mice live longer than PSDCKO mice, most likely due to a milder skin phenotype. (B) Monitoring WBC count shows that B-LPD, although delayed, surges in N1N2CKO and PSDCKO mice receiving sublethal or focal irradiation. (1.1 MB JPG) [file pbio.0060123.sg004.jpg]

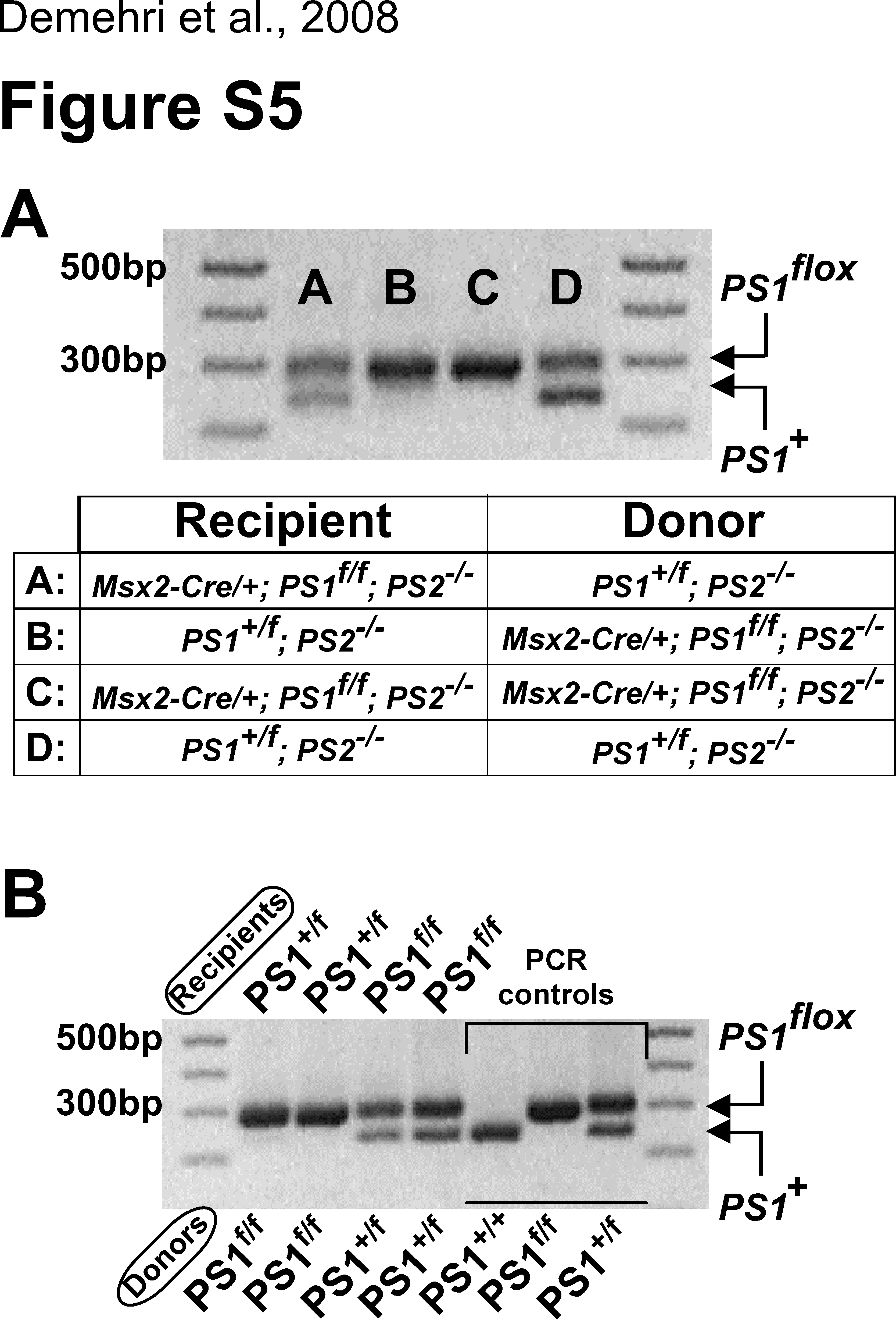

Supplement: Figure S5 — DNA from peripheral blood of recipient animals analyzed for the presence of PS1 alleles 25 d after BMT confirms repopulation of the recipients' hematopoietic system by donor-derived BM cells in both (A) rescue and (B) disease propagation experiments. Because there is no Cre activity in the hematopoietic system, the difference between homozygous (one band) and heterozygous (two bands) is detected. Controls for the various genotypes are shown in (B). (2.1 MB JPG) [file pbio.0060123.sg005.jpg]

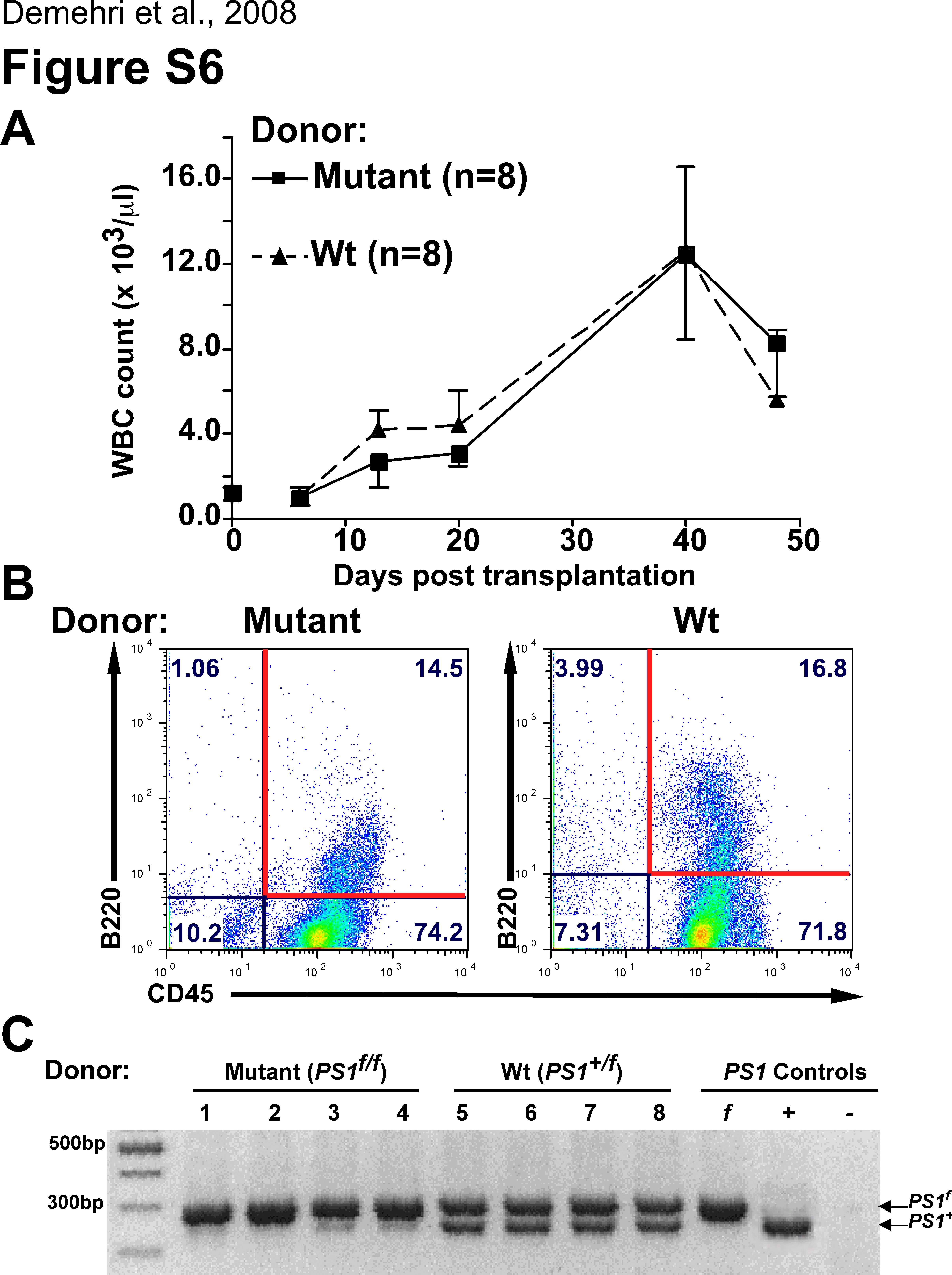

Supplement: Figure S6 — (A) NOD/SCID mice receiving BM from mutant or wild-type newborn donors are indistinguishable over 2 mo of follow-up. (B) The percentage of B220+ B cells in mice receiving BM from mutant or wild-type donors is not significantly different. (C) Genotyping for PS1 alleles in peripheral blood from NOD/SCID mice 25 days after BMT shows that most of their WBCs are derived from the donor BM. (3.3 MB JPG) [file pbio.0060123.sg006.jpg]

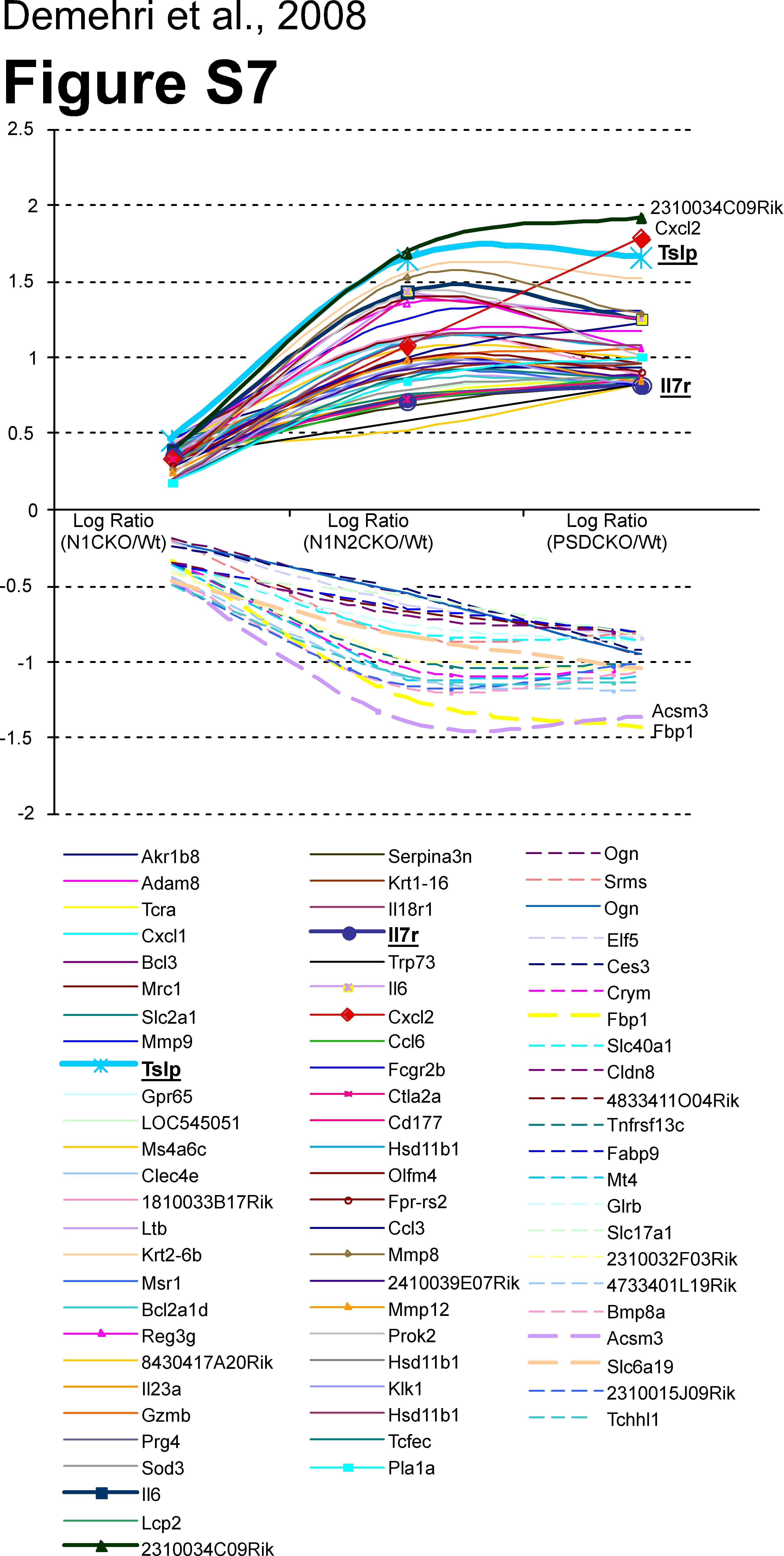

Supplement: Figure S7 — Cytokines/chemokines that are up- or down-regulated in P9 mutant versus wild-type skin mRNA samples are enriched by this analysis. Note the absence of TNFα. (2.7 MB JPG) [file pbio.0060123.sg007.jpg]

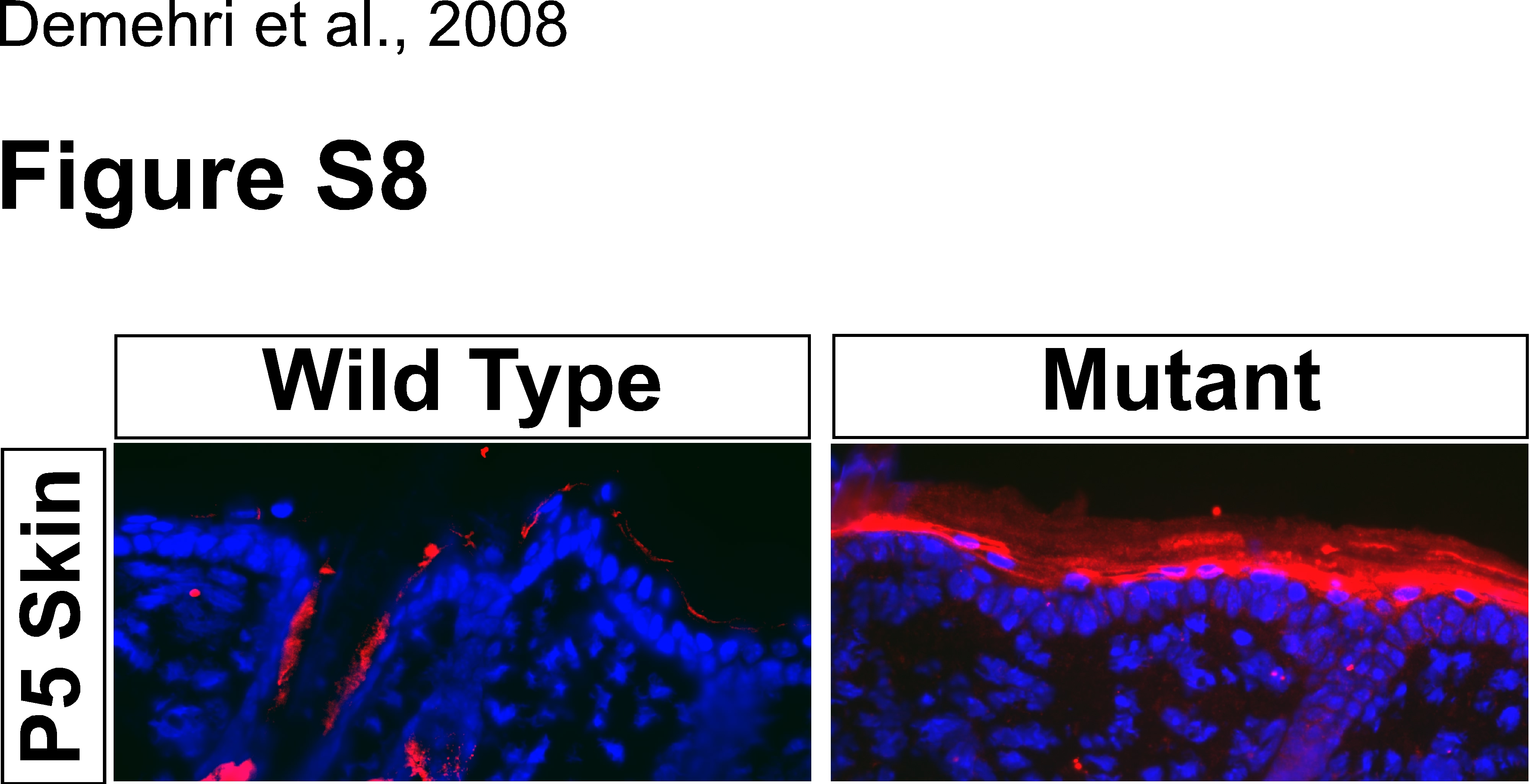

Supplement: Figure S8 — PSDCKO mice produce TSLP in suprabasal keratinocytes whereas wild-type mice do not (dashed lines demarcate the basement membrane; 200× magnification). For immunoflorescence, Cy-3-conjugated streptavidin is used to detect biotinylated α-TSLP, and sections are counterstained with DAPI nuclear stain. (729 KB JPG) [file pbio.0060123.sg008.jpg]

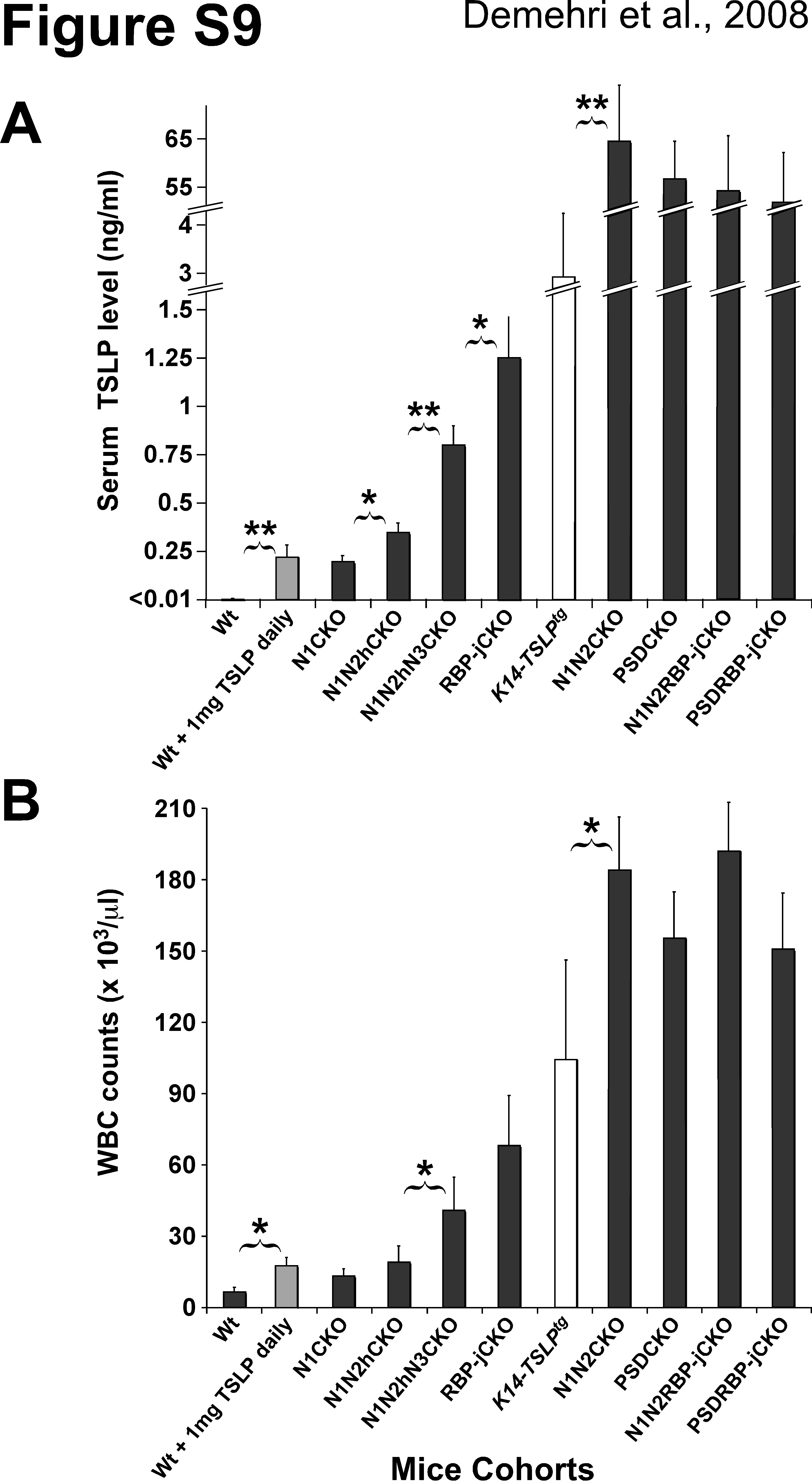

Supplement: Figure S9 — Comparing (A) the serum TSLP levels and (B) WBC counts of all animal cohorts examined in this study (compiled from the data shown in Figure 1, 5, and 6) reveals a tight correlation between the systemic TSLP elevation and WBC count increase in newborn mice. The measurements are obtained from mutant mice in the second week of life and from TSLP-treated wild-type animals at P8 (n = 3, for each group; significant difference between adjacent genotypes is highlighted: *, p < 0.05, **, p < 0.01). (1.3 MB JPG) [file pbio.0060123.sg009.jpg]

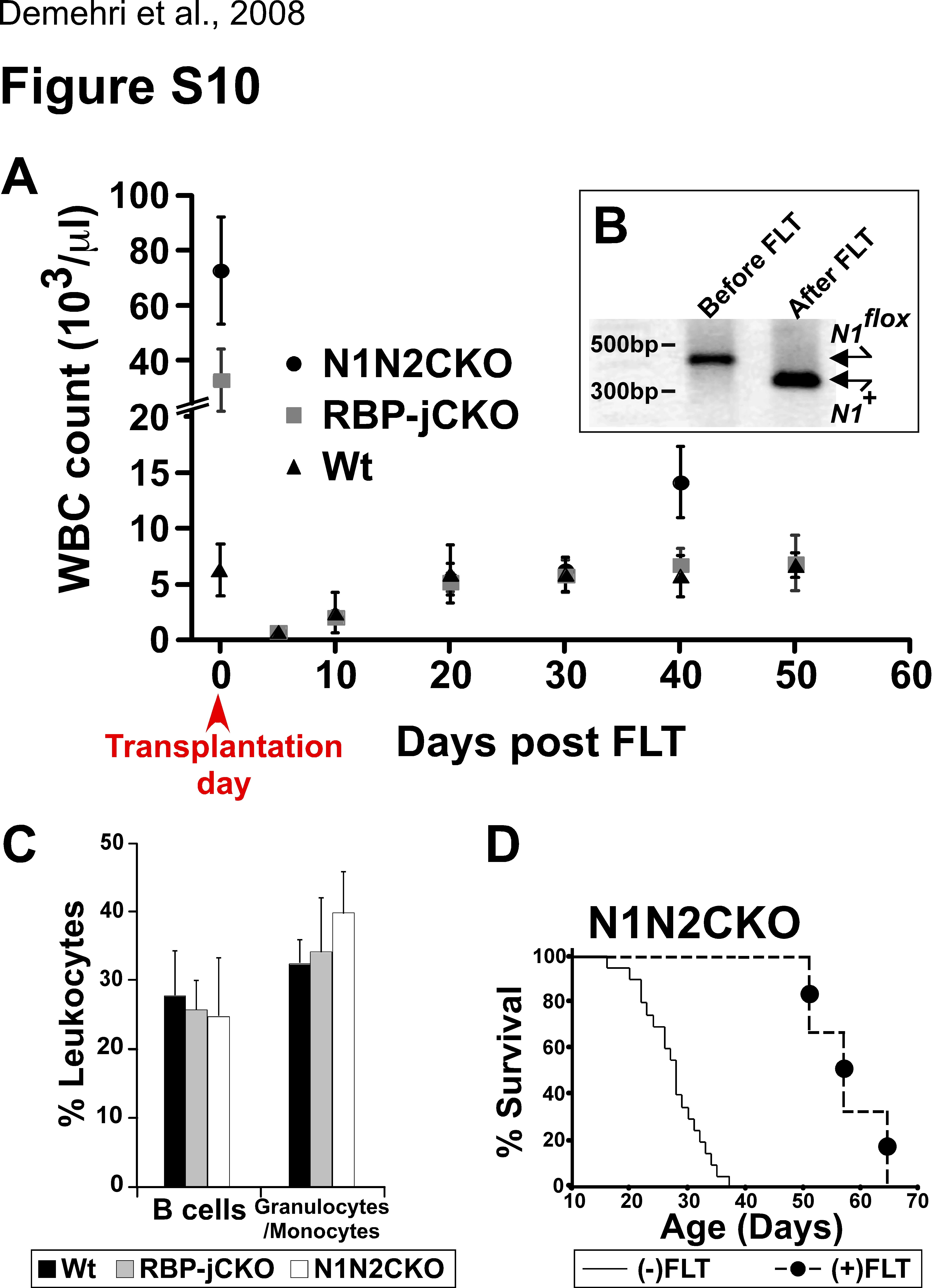

Supplement: Figure S10 — (A) Monitoring the WBC counts of the transplanted mice shows no B-LPD recurrence in the mutant animals after FLT (n = 3, for each group). (B) DNA from peripheral blood of transplanted N1N2CKO animals, analyzed for the presence of Notch1 alleles 25 days after FLT, confirms the repopulation of the recipients' hematopoietic system by donor-derived fetal liver cells. (C) FC analysis on peripheral blood from transplanted N1N2CKO, RBP-jCKO, and wild-type mice collected 25 days after FLT shows no sign of B cell expansion in the mutant animals. (D) As with BMT, curing lethal B-LPD in N1N2CKO animals by FLT (n = 3) leads to a relative extension in life span compared to untransplanted N1N2CKO mice (n = 20; p < 0.001, log rank test). (1.5 MB JPG) [file pbio.0060123.sg010.jpg]

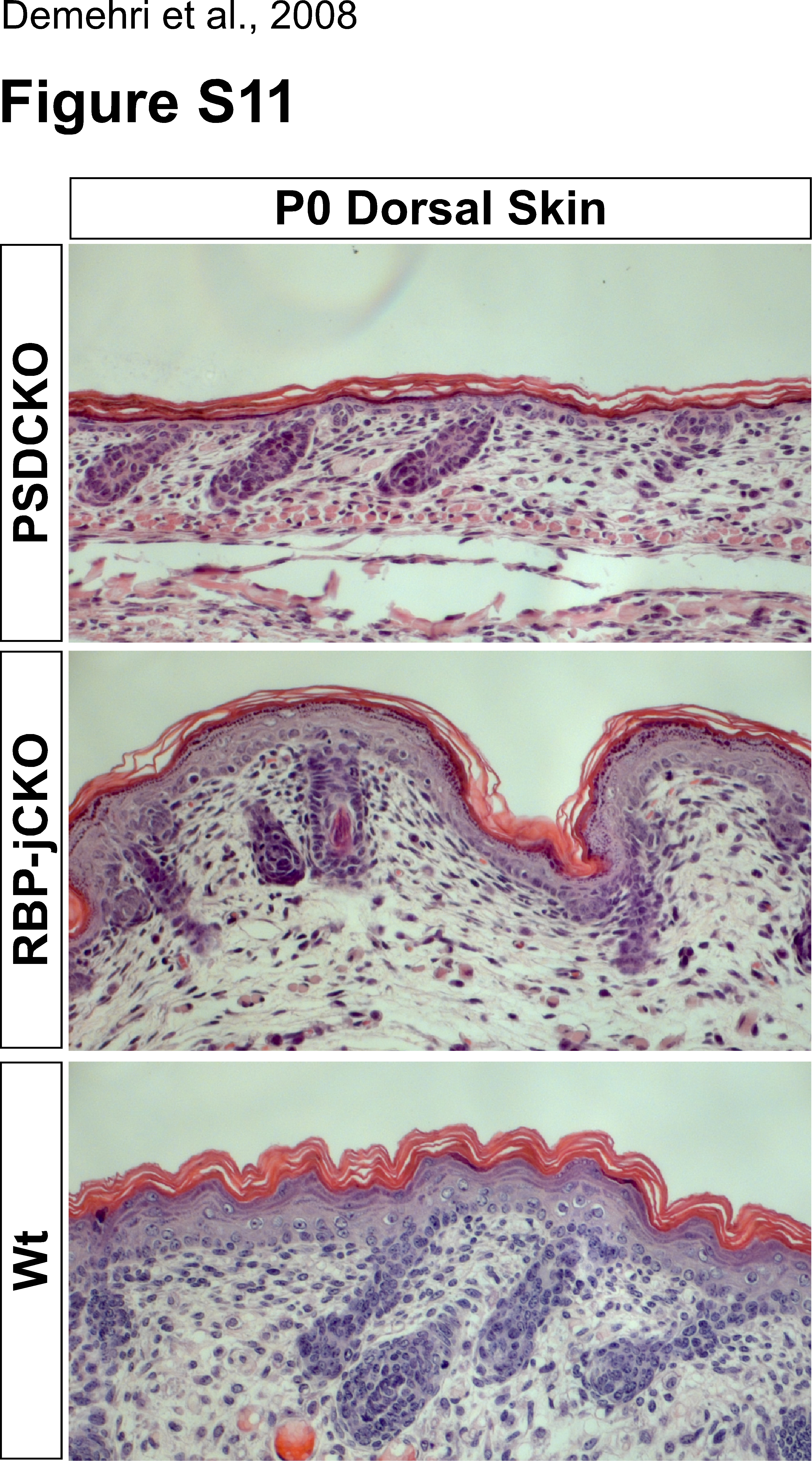

Supplement: Figure S11 — Cross-sections of the P0 dorsal skin from PSDCKO, RBP-jCKO, and wild-type mice show a progressive loss of epidermal differentiation (200× magnification). PSDCKO skin is most severely affected with loss of all epidermal layers other than basal and squamous. A less severe defect is seen in RBP-jCKO skin, correlating with the lower TSLP levels and improved survival of RBP-jCKO compared to PSDCKO animals. (4.3 MB JPG) [file pbio.0060123.sg011.jpg]
