# Supplementary material for: Notch-Deficient Skin Induces a Lethal Systemic B-Lymphoproliferative Disorder by Secreting TSLP, a Sentinel for Epidermal Integrity
Source: PLoS Biol. 2008 May 27;6(5):e123. doi: 10.1371/journal.pbio.0060123 (PMC2430908; doi:10.1371/journal.pbio.0060123)
Supplement: Table S1 — Measuring the weight of the animals and their organs at P14 reveals that mutant mice are smaller than their wild-type littermates yet have larger spleen and lymph nodes (n = 3, for each group). The values are presented as mean ± standard deviation (“a” indicates p < 0.001 (compared to wild-type control)). (35 KB PDF) [file pbio.0060123.st001.pdf]

Demehri et al., 2008

Table S1

Genotype	Weight (g)	Spleen mass (g)	Liver mass (g)	Lymph Nodes
PSDCKO	6.8 $\pm$ 2.0	0.22 $\pm$ 0.03[a]	0.53 $\pm$ 0.05	Enlarged
N1N2CKO	6.6 $\pm$ 2.2	0.20 $\pm$ 0.03[a]	0.56 $\pm$ 0.04	Enlarged
Wt	10.6 $\pm$ 0.6	0.08 $\pm$ 0.02	0.63 $\pm$ 0.09	Normal
