# Supplementary material for: Notch-Deficient Skin Induces a Lethal Systemic B-Lymphoproliferative Disorder by Secreting TSLP, a Sentinel for Epidermal Integrity
Source: PLoS Biol. 2008 May 27;6(5):e123. doi: 10.1371/journal.pbio.0060123 (PMC2430908; doi:10.1371/journal.pbio.0060123)
Supplement: Table S2 — Values are shown as log of fold change. Down-regulated genes are labeled with a minus sign. (44 KB PDF) [file pbio.0060123.st002.pdf]

Demehri et al., 2008

Table S2

Gene Symbol	Gene Title	Log(Ratio) N1CKO/Wt	Log(Ratio) N1N2CKO/Wt	Log(Ratio) PSDCKO/Wt
2310034C09Rik	RIKEN cDNA 2310034C09 gene	0.38	1.688	1.92
Cxcl2	chemokine (C-X-C motif) ligand 2	0.335	1.076	1.782
Tslp	thymic stromal lymphopoietin	0.46	1.64	1.662
Krt2-6b	keratin complex 2, basic, gene 6g	0.424	1.557	1.516
Mmp9	matrix metalloproteinase 9	0.461	1.261	1.306
Mmp8	matrix metalloproteinase 8	0.26	1.523	1.29
Il6	interleukin 6	0.391	1.43	1.255
Il6	interleukin 6	0.346	1.43	1.255
1190003K14Rik	CD177 antigen	0.317	1.398	1.254
Ccl3	chemokine (C-C motif) ligand 3	0.281	0.998	1.226
Adam8	a disintegrin and metalloprotease domain 8	0.484	1.142	1.17
Hsd11b1	11 beta-hydroxysteroid dehydrogenase-1	0.315	1.099	1.075
Hsd11b1	11 beta-hydroxysteroid dehydrogenase-1	0.205	1.099	1.075
Hsd11b1	11 beta-hydroxysteroid dehydrogenase-1	0.193	1.099	1.075
Cxcl1	chemokine (C-X-C motif) ligand 1	0.477	1.122	1.064
Clec4e	C-type lectin domain family 4, member e	0.439	1.123	1.061
Reg3g	regenerating islet-derived 3 gamma	0.416	1.358	1.06
Gzmb	granzyme B	0.405	0.972	1.055
Olfm4	olfactomedin 4	0.31	1.384	1.019
Prok2	prokineticin 2	0.228	1.418	1.014
Pla1a	phospholipase A1 member A	0.182	0.85	1.003
Ms4a6c	membrane-spanning 4-domains, subfamily A, member 6C	0.443	1.055	0.999
Mrc1	mannose receptor, C type 1	0.469	1.133	0.955
Prg4	proteoglycan 4	0.405	0.939	0.955
Krt1-16	keratin complex 1, acidic, gene 16	0.363	0.898	0.955
Gpr65	G-protein coupled receptor 25	0.458	0.962	0.953
Akr1b8	aldo-keto reductase family 1, member B8	0.484	0.889	0.94
Fpr-rs2	Mus musculus N-formylpeptide receptor-like 2 gene	0.301	0.992	0.912
1810033B17Rik	RIKEN cDNA 1810033B17 gene	0.434	1.145	0.895
Tcra	2 days neonate thymus thymic cells cDNA, RIKEN full-length enriched library	0.482	0.751	0.893
Bcl3	B-cell leukemia/lymphoma 3	0.475	0.788	0.891
LOC545051	similar to T-cell receptor alpha chain C region	0.458	0.782	0.88
Ccl6	chemokine (C-C motif) ligand 6	0.332	0.72	0.879
Sod3	superoxide dismutase 3, extracellular	0.394	0.79	0.878
2410039E07Rik	RIKEN cDNA 2410039E07 gene	0.252	0.923	0.877
Lcp2	lymphocyte cytosolic protein 2	0.385	0.971	0.864
Tcfec	transcription factor EC	0.185	0.873	0.863
Ctla2a	trophoblast specific protein beta	0.327	0.723	0.861
Il23a	interleukin 23, alpha subunit p19	0.415	0.752	0.86
Mmp12	matrix metalloproteinase 12	0.251	0.976	0.847
Serpina3n	serine (or cysteine) proteinase inhibitor, clade A, member 3N	0.376	0.685	0.842
Ltb	lymphotoxin B	0.432	0.847	0.841
Slc2a1	solute carrier family 2 (facilitated glucose transporter), member 1	0.469	0.753	0.838
Klk1	kallikrein 9	0.194	0.951	0.824
Bcl2a1d	B-cell leukemia/lymphoma 2 related protein A1a	0.421	0.852	0.823
8430417A20Rik	RIKEN cDNA 8430417A20 gene	0.416	0.528	0.823
Il18r1	interleukin 18 receptor 1	0.358	0.743	0.821
Trp73	Mus musculus p73 gene, exons 5 through 14 and partial cds.	0.35	0.586	0.821
Fcgr2b	Mouse beta Fc receptor type II (FCRII) gene, complete cds.	0.328	0.982	0.819
Il7r	interleukin 7 receptor	0.351	0.718	0.815
Msr1	macrophage scavenger receptor 1	0.423	0.898	0.81
Fabp9	fatty acid binding protein 9, testis	-0.359	-0.648	-0.805
4833411O04Rik	RIKEN cDNA 2600010E01 gene	-0.35	-0.672	-0.807
Srms	src-related kinase lacking C-terminal regulatory tyrosine and N-terminal myristylation sites	-0.202	-0.831	-0.808
Cldn8	claudin 8	-0.347	-0.695	-0.808
Slc17a1	solute carrier family 17 (sodium phosphate), member 1	-0.41	-0.566	-0.823
Elf5	E74-like factor 5	-0.215	-0.627	-0.848
Slc40a1	solute carrier family 40 (iron-regulated transporter), member 1	-0.343	-0.801	-0.851
Glrb	glycine receptor, beta subunit	-0.387	-0.753	-0.865
Ces3	carboxylesterase 3	-0.235	-0.52	-0.924
Ogn	osteoglycin	-0.189	-0.552	-0.952
Ogn	osteoglycin	-0.214	-0.552	-0.952
2310015J09Rik	RIKEN cDNA 2310015J09 gene	-0.492	-1.156	-1.008
Tnfrsf13c	tumor necrosis factor receptor superfamily, member 13c	-0.353	-0.989	-1.018
Slc6a19	solute carrier family 6 (neurotransmitter transporter), member 19	-0.479	-0.833	-1.05
Crym	crystallin, mu	-0.338	-1.043	-1.052
2310032F03Rik	RIKEN cDNA 2310032F03 gene	-0.41	-0.992	-1.061
Bmp8a	3-oxoacid CoA transferase 2A	-0.449	-1.172	-1.068
Mt4	metallothionein 4	-0.366	-1.118	-1.101
Tchhl1	trichohyalin-like 1	-0.498	-1.09	-1.131
4733401L19Rik	RIKEN cDNA 4733401L19 gene	-0.446	-1.101	-1.19
Acsm3	acyl-CoA synthetase medium-chain family member 3	-0.473	-1.396	-1.37
Fbp1	fructose bisphosphatase 1	-0.343	-1.237	-1.434
