# Supplementary material for: Notch-Deficient Skin Induces a Lethal Systemic B-Lymphoproliferative Disorder by Secreting TSLP, a Sentinel for Epidermal Integrity
Source: PLoS Biol. 2008 May 27;6(5):e123. doi: 10.1371/journal.pbio.0060123 (PMC2430908; doi:10.1371/journal.pbio.0060123)
Supplement: Table S3 — Comprehensive (A) antigen and (B) autoimmune analysis on sera collected from P14 mutant (N1N2CKO; n = 3) and wild-type (n = 3) animals does not identify any other factor besides TSLP that could potentially explain B-LPD in the mutant animals. The antigens with significantly higher levels in mutant serum are marked (values in red). Note that the least detectable dose for each factor is calculated as three standard deviations above the average value obtained measuring 20 blank samples. Therefore, values below the least detectable dose are below accurate detection range. Rules Based Medicine laboratory cutoff values for autoimmune analysis signify the upper limits of normal ratios for each factor measured. (46 KB PDF) [file pbio.0060123.st003.pdf]

Demehri et al., 2008

Table S3

A

Antigen:		Apo A1 (Apolipoprotein A1)	Beta-2 Microglobulin	Calbindin	CD40	CD40 Ligand	Clusterin	CRP (C Reactive Protein)	Cystatin-C	EGF (Epidermal Growth Factor)	Endothelin-1	Eotaxin	Factor VII
		ug/mL	ug/mL	ng/mL	pg/mL	pg/mL	ug/mL	ug/mL	ng/mL	pg/mL	pg/mL	pg/mL	ng/mL
Least Detectable Dose:		10	0.89	0.043	12	92	0.55	0.83	<9.9	39	67	12	0.96
Samples:	Mutant	45	<LOW>	104	668	713	125	1.3	2000	16	35	831	0.97
(Average Value)	Wild Type	50	<LOW>	40	542	639	76	1.5	1250	13	30	774	0.81

Antigen:		FGF-9 (Fibroblast Growth Factor-9)	FGF-basic (Fibroblast Growth Factor-basic)	Fibrinogen	GCP-2 (Granulocyte Chemotactic Protein-2)	GM-CSF (Granulocyte Macrophage-Colony Stimulating Factor)	Growth Hormone	GST-alpha (Glutathione S-Transferase alpha)	GST-Mu	Haptoglobin	IFN-gamma (Interferon-gamma)	IgA (Immunoglobulin A)	IL-10 (Interleukin-10)
		ng/mL	ng/mL	ug/mL	ng/mL	pg/mL	ng/mL	ng/mL	ng/mL	ug/mL	pg/mL	ug/mL	pg/mL
Least Detectable Dose:		0.99	0.58	170	0.025	8.7	4.2	0.42	3.0	0.64	68	1.9	109
Samples:	Mutant	<LOW>	2.7	224	28	<LOW>	25	<LOW>	<LOW>	93	<LOW>	6.7	432
(Average Value)	Wild Type	<LOW>	1.9	60	21	<LOW>	36	<LOW>	<LOW>	26	<LOW>	6.7	412

Antigen:		IL-11 (Interleukin-11)	IL-12p70 (Interleukin-12p70)	IL-17 (Interleukin-17)	IL-18 (Interleukin-18)	IL-1alpha (Interleukin-1alpha)	IL-1beta (Interleukin-1beta)	IL-2 (Interleukin-2)	IL-3 (Interleukin-3)	IL-4 (Interleukin-4)	IL-5 (Interleukin-5)	IL-6 (Interleukin-6)	IL-7 (Interleukin-7)
		pg/mL	ng/mL	ng/mL	ng/mL	pg/mL	ng/mL	pg/mL	pg/mL	pg/mL	ng/mL	pg/mL	ng/mL
Least Detectable Dose:		87	0.57	0.15	0.67	45	0.45	67	21	74	0.19	14	0.03
Samples:	Mutant	<LOW>	<LOW>	<LOW>	1.5	7400	1.8	<LOW>	<LOW>	<LOW>	0.69	<LOW>	<LOW>
(Average Value)	Wild Type	<LOW>	<LOW>	<LOW>	1.1	1620	1.5	<LOW>	<LOW>	<LOW>	0.43	<LOW>	<LOW>

Antigen:		Insulin	IP-10 (Inducible Protein-10)	KC/GROalpha (Melanoma Growth Stimulatory Activity Protein)	Leptin	LIF (Leukemia Inhibitory Factor)	Lymphotactin	MCP-1 (Monocyte Chemoattractant Protein-1)	MCP-3 (Monocyte Chemoattractant Protein-3)	MCP-5 (Monocyte Chemoattractant Protein-5)	M-CSF (Macrophage-Colony Stimulating Factor)	MDC (Macrophage-Derived Chemokine)	MIP-1alpha (Macrophage Inflammatory Protein-1alpha)
		uIU/mL	pg/mL	ng/mL	ng/mL	pg/mL	pg/mL	pg/mL	pg/mL	pg/mL	ng/mL	pg/mL	ng/mL
Least Detectable Dose:		1.0	40	0.17	0.096	44	85	17	31	46	0.018	22	0.23
Samples:	Mutant	1.4	95	<LOW>	1.1	358	239	591	1740	151	6.8	2020	0.74
(Average Value)	Wild Type	1.0	123	<LOW>	3.8	252	163	166	371	31	8.1	735	0.32

Antigen:		MIP-1beta (Macrophage Inflammatory Protein-1beta)	MIP-1gamma (Macrophage Inflammatory Protein-1gamma)	MIP-2 (Macrophage Inflammatory Protein-2)	MIP-3beta (Macrophage Inflammatory Protein-3beta)	MMP-9 (Matrix Metalloproteinase-9)	MPO (Myeloperoxidase)	Myoglobin	NGAL	OSM (Oncostatin M)	Osteopontin	RANTES (Regulation Upon Activation, Normal T-Cell Expressed and Secreted)	SAP (Serum Amyloid P)
		pg/mL	ng/mL	pg/mL	ng/mL	ng/mL	ng/mL	ng/mL	ng/mL	ng/mL	ng/mL	pg/mL	ug/mL
Least Detectable Dose:		78	0.074	7.2	0.47	10	0.95	24	5.8	0.13	<4.0	48	5.4
Samples:	Mutant	90	26	26	<LOW>	624	185	35	<LOW>	0.097	720	<LOW>	16
(Average Value)	Wild Type	56	11	26	<LOW>	153	58	16	<LOW>	0.026	498	<LOW>	18

Antigen:		SCF (Stem Cell Factor)	SGOT (Serum Glutamic-Oxaloacetic Transaminase)	TIMP-1 (Tissue Inhibitor of Metalloproteinase Type-1)	Tissue Factor	TNF-alpha (Tumor Necrosis Factor-alpha)	TPO (Thrombopoietin)	VCAM-1 (Vascular Cell Adhesion Molecule-1)	VEGF (Vascular Endothelial Cell Growth Factor)	vWF (von Willebrand Factor)	TSLP (thymic stromal lymphopoietin)
		pg/mL	ug/mL	ng/mL	ng/mL	ng/mL	ng/mL	ng/mL	pg/mL	ng/mL	pg/mL
Least Detectable Dose:		75	1.9	0.18	0.52	0.14	2.7	19	38	99	10
Samples:	Mutant	295	<LOW>	14	2.5	0.055	15	2490	428	131	52000
(Average Value)	Wild Type	218	<LOW>	4.3	2.9	0.037	15	1800	290	141	<LOW>

B

Autoimmune Serology:		Beta-2 Glycoprotein Antibody	Insulin Antibody	Jo-1 (histidyl-RNA synthetase) Antibody	Mitochondrial Antibody	MPO (Myeloperoxidase) Antibody	pCNA (Proliferating Cell Nuclear Antigen) Antibody	Proteinase 3 Antibody	Ribosomal P Antibody	RNP (Ribonuclear Protein) Antibody	SCL-70 (Scleroderma-70) Antibody	Smith Antibody	SSA (Sjogren's Syndrome A) Antibody	SSB (Sjogren's Syndrome B) Antibody
		RBM Ratio	RBM Ratio	RBM Ratio	RBM Ratio	RBM Ratio	RBM Ratio	RBM Ratio	RBM Ratio	RBM Ratio	RBM Ratio	RBM Ratio	RBM Ratio	RBM Ratio
RBM Mouse Cutoff:		7	2.60	1.100	4	4	2.20	2.50	2.20	6	6	2	0.77	2.30
Samples:	Mutant	2	0.96	1	0	0	1	1.0	1	2	2	1	0.00	0.85
(Average value)	Wild Type	1	0.77	0	1	0	1	0.9	0	1	1	1	-1.25	0.85
