# Supplementary material for: Notch-Deficient Skin Induces a Lethal Systemic B-Lymphoproliferative Disorder by Secreting TSLP, a Sentinel for Epidermal Integrity
Source: PLoS Biol. 2008 May 27;6(5):e123. doi: 10.1371/journal.pbio.0060123 (PMC2430908; doi:10.1371/journal.pbio.0060123)
Supplement: Table S4 — For comparison, TSLP probes also are included. The color highlights samples with p < 0.005. (59 KB PDF) [file pbio.0060123.st004.pdf]

Demehri et al., 2008

# Table S4

| Probe ID | Symbol | Definition | *p* -value (PSDCKO/Wt) | GFoldChange (PSDCKO/Wt) | Log(ratio) (PSDCKO/Wt) |
|---|---|---|---|---|---|
| 6290093 | Alox12 | arachidonate 12-lipoxygenase (Alox12). | **0.000001** | **-19.03** | **-1.279** |
| 4150397 | Asah3 | N-acylsphingosine amidohydrolase (alkaline ceramidase) 3 (Asah3). | **0.000001** | **-6.96** | **-0.843** |
| 2060064 | Alox12e | arachidonate lipoxygenase, epidermal (Alox12e). | **0.000176** | **-5.75** | **-0.760** |
| 3170427 | Adh1 | alcohol dehydrogenase 1 (class I) (Adh1). | **0.001750** | **-3.37** | **-0.528** |
| 5340746 | Elovl6 | ELOVL family member 6, elongation of long chain fatty acids (yeast) (Elovl6). | **0.001571** | **-2.93** | **-0.467** |
| 2360309 | Liph | lipase, member H (Liph). | **0.001263** | **-2.55** | **-0.407** |
| 2630372 | Aloxe3 | arachidonate lipoxygenase 3 (Aloxe3). | **0.001286** | **-2.32** | **-0.365** |
| 101510348 | Alox12 | arachidonate 12-lipoxygenase (Alox12). | **0.000002** | **-2.21** | **-0.345** |
| 2690050 | Liph | lipase, member H (Liph). | **0.009203** | **-2.19** | **-0.341** |
| 2680441 | Scd1 | stearoyl-Coenzyme A desaturase 1 (Scd1) | 0.017313 | -2.19 | -0.340 |
| 4760138 | Elovl1 | elongation of very long chain fatty acids (FEN1/Elo2, SUR4/Elo3, yeast)-like 1 (Elovl1). | **0.005893** | **-2.03** | **-0.308** |
| 105360215 | Lipl3 | lipase-like, ab-hydrolase domain containing 3 | **0.000253** | **-1.91** | **-0.280** |
| 3930707 | Sgpp1 | sphingosine-1-phosphate phosphatase 1 (Sgpp1). | **0.000093** | **-1.85** | **-0.267** |
| 730193 | Ptgs1 | prostaglandin-endoperoxide synthase 1 (Ptgs1). | **0.003103** | **-1.63** | **-0.213** |
| 6450195 | Ptgs1 | prostaglandin-endoperoxide synthase 1 (Ptgs1). | **0.001999** | **-1.46** | **-0.164** |
| 1780273 | Ptgs1 | prostaglandin-endoperoxide synthase 1 (Ptgs1). | 0.021620 | -1.43 | -0.155 |
| 1410309 | Smpd1 | sphingomyelin phosphodiesterase 1, acid lysosomal (Smpd1). | 0.111435 | -1.39 | -0.142 |
| 5220333 | Elovl4 | elongation of very long chain fatty acids (FEN1/Elo2, SUR4/Elo3, yeast)-like 4 (Elovl4). | 0.625118 | -1.07 | -0.028 |
| 6590059 | Lipl2 | lipase-like, ab-hydrolase domain containing 2 | 0.814034 | -1.06 | -0.027 |
| 6860050 | Elovl3 | elongation of very long chain fatty acids (FEN1/Elo2, SUR4/Elo3, yeast)-like 3 (Elovl3). | 0.827319 | -1.05 | -0.022 |
| 2450019 | Elovl1 | elongation of very long chain fatty acids (FEN1/Elo2, SUR4/Elo3, yeast)-like 1 (Elovl1). | 0.899655 | -1.03 | -0.012 |
| 70458 | Lipl2 | lipase-like, ab-hydrolase domain containing 2 | 0.629916 | -1.03 | -0.012 |
| 2370358 | Tslpr | thymic stromal-derived lymphopoietin, receptor (Tslpr). | 0.831755 | -1.01 | -0.004 |
| 1990500 | Tslp | thymic stromal lymphopoietin (Tslp). | 0.980370 | 1.00 | 0.001 |
| 5550717 | Smpd1 | sphingomyelin phosphodiesterase 1, acid lysosomal (Smpd1). | 0.841169 | 1.04 | 0.017 |
| 106200484 | Elovl3 | elongation of very long chain fatty acids (FEN1/Elo2, SUR4/Elo3, yeast)-like 3 (Elovl3). | 0.191433 | 1.06 | 0.025 |
| 4810181 | Sult2b1 | sulfotransferase family, cytosolic, 2B, member 1 (Sult2b1). | 0.713639 | 1.07 | 0.028 |
| 520164 | Smpd3 | sphingomyelin phosphodiesterase 3, neutral (Smpd3). | 0.491531 | 1.16 | 0.063 |
| 3360484 | Sult2b1 | sulfotransferase family, cytosolic, 2B, member 1 (Sult2b1). | 0.124677 | 1.50 | 0.177 |
| 730408 | Tslp | thymic stromal lymphopoietin (Tslp). | **0.000116** | 19.79 | **1.296** |
