# Supplementary material for: Notch-Deficient Skin Induces a Lethal Systemic B-Lymphoproliferative Disorder by Secreting TSLP, a Sentinel for Epidermal Integrity
Source: PLoS Biol. 2008 May 27;6(5):e123. doi: 10.1371/journal.pbio.0060123 (PMC2430908; doi:10.1371/journal.pbio.0060123)
Supplement: Text S1 — (54 KB DOC) [file pbio.0060123.sd001.doc]

## Text S1

## Supplemental Material and Methods

Sublethal and focal irradiation.

In the sublethal irradiation experiments, mutant and wild-type newborns (~P10) received a sublethal dose of total body irradiation (450-cGy)[1]. The thymus or liver of the newborn animals was focally irradiated by delivering 950-cGy of γ-irradiation to a ~20mm^2^ area of the skin over the respective organ by using a microRT® radiation device[2]. Irradiation was specifically targeted to thymus or liver. The organ-targeted irradiation did not destroy the host hematopoiesis; therefore, no post-irradiation BMT was required.

**Fetal liver transplantation (FLT).**

N1N2CKO, RBP-jCKO and their wild-type littermates (age: P8-P12) were lethally irradiated with 950-cGy, and injected intravenously with 2x10^6^ fetal liver cells from E13.5 CD1 wild-type embryos in 100ml PBS+2%FBS.

**Serology.**

For comprehensive serum antigen and autoimmune signature measurements, serum samples were collected, frozen and shipped on dry ice to Rules Based Medicine Laboratory (Austin, TX).

**PCR and qRT-PCR primer sequences.**

Primers used to amplify the wild-type and floxed *PS1* allele were: 5’-acgtggcaggggcctcaactcctccagagtcagg-3’ and 5’-ttttggtctctttctgccccctctccattttctc-3’; the former primer was used in conjunction with primer 5’-ctggccttgtcaaggtttccctccatcttggttg-3’ to amplify the Cre-mediated deleted *PS1* allele (*PS1^Δ^*. The primers used to amplify TSLP were: 5’-CCAGGCTACCCTGAAACTGA-3’ and 5’-TCTGGAGATTGCATGAAGGA-3’; the primers used for IL-7 are: 5’-ATCCTTGTTCTGCTGCCTGT-3’ and 5’-TGGTTCATTATTCGGGCAAT-3’.

RNA isolation and microarray analyses.

For total skin microarray analyses, total dorsal skin RNA was collected from five P9 mice of each genotype (N1CKO, N1N2CKO, PSDCKO and their wild-type littermates) using QIAGEN RNeasy Mini Kit (QIAGEN Sciences, Valencia, CA). cRNA was prepared for the hybridization conducted at Rosetta Inpharmatics (Seattle, WA). Both Resolver microarray suite (MAS) and dChip software <[http://www.dchip.org](http://www.dchip.org/)> were used for analysis and comparisons. 1176 genes (approximately 5% of the genes on the array) were altered more than 1.5-fold at *p* <0.0001 in all three the mutant groups we analyzed. For reverse trend analysis, fold change values were converted to Log10 values, imported into excel an algorithm scored the data for systematic increase in fold value as Notch functions diminish. Only 73 genes fit the search criteria.

To monitor transcriptional changes within the epidermis, epidermal microarray analysis was performed on mRNA samples from dorsal epidermal keratinocytes of three PSDCKO mice and their wild-type littermates at P9. Keratinocytes were isolated by scraping them off frozen skin on dry ice. RNAs purified by using QIAGEN RNeasy Mini Kit (QIAGEN Sciences) were qualitatively assessed by RNA BioAnalyzer2100 (Agilent, Palo Alto, CA) and quantified spectrophotometrically in NanoDrop ND-1000 (NanoDrop Technologies, Wilmington, DE). For each genotype, labeled aRNA derived from 100ng of total RNA was hybridized to an array of the Illumina Mouse6 expression beadchip according to Illumina’s protocol.  Three samples of each genotype were hybridized to three arrays at the Microarray Facility at Washington University Genome Sequencing Center <<http://genome.wustl.edu/services/microarray.cgi>>. After the arrays were scanned, image data was de-encrypted using Illumina BeadStudio.  Non-normalized intensity values were imported into Partek Genomics Suite (St. Louis, MO).  Data was first quantile normalized then log2 normalized. ANOVA analysis was used to determine significant differences between groups, removing the small contribution of litter-to-litter and chip-to-chip variation. Lipid metabolic enzyme heat map was generated based on a list of enzymes involved in lipid synthesis pathway[3], using Spotfire^TM^ software.

To analyze *wrfr-/-* RNA, total RNAs were isolated from dorsal skin of *wrfr-/- and wrfr+/-* embryos at E15.5 using a tissue homogenizer (Powergen 125) and the RNeasy Fibrous Tissue Mini kit following the manufacturer’s instructions (Qiagen, Chatsworth, CA). Tail tips were saved for genotyping by PCR. The purified RNAs were qualitatively assessed by RNA LabChip (Agilent) and quantified spectrophotometrically in NanoDrop ND-1000 (NanoDrop Technologies). To make analytical duplicates, equal amounts of two RNA samples of the same genotype were pooled and split into two tubes. The preparation of biotin-labeled, antisense cRNA targets and microarray hybridization were performed in the Gene Chip Facility in campus (Alvin J. Siteman Cancer Center, Washington University) using the standard protocols supplied by the manufacturer (Affymetrix, Santa Clara, CA). Double-stranded cDNAs were generated from 5µg of total RNA with T7-T24 primers (Genset, Paris, France), Superscript II reverse transcriptase, E. coli ligase, and E. coli polymerase I (Invitrogen, Carlsbad, CA). Biotin-labeled, antisense cRNA targets were generated from the synthesized cDNAs using the Enzo BioArray High Yield RNA Transcript Labeling kit (Enzo Biochemical, New York, NY). Ten micrograms of each biotinylated cRNA preparation were fragmented and hybridized to the mouse genome array MOE430A2 for 16hr in Hybridization Oven 640. The data were processed using Affymetrix Microarray Suite (MAS 5.0, Affymetrix Inc., Santa Clara, CA) and exported as text files containing both qualitative and quantitative analyses for each probe set. The cell intensity files were normalized based on total intensity using affy package (RMA method) from BioConductor ([http://www.bioconductor.org](http://www.bioconductor.org/), Biostatistics Unit of Dana Farber Cancer Institute, Harvard Medical School/ Harvard School of Public Health) and exported as text files for numerical analyses. *Wrfr* embryos with or without gross skin abnormalities (experimental samples as the numerator) were compared to their heterozygous littermates (control samples as the denominator). Data from analytical duplicates were analyzed statistically by multtest package (Resampling-based multiple hypothesis testing, BioConductor) and those with adjusted p-value less than 0.05 were selected. To treat up- and down-regulated genes in a similar fashion, the fold changes were indicated in logarithm base 2. To filter false positives, data with increased fold changes and with absent calls in the experimental samples or those with decreased fold changes and with absent calls in the control samples were eliminated. A cut-off value of 0.59 in logarithm base 2, i.e., 1.5-fold change, was used to identify genes that show most significant variation in expression. To biologically annotate genes that show differential expression, GenMAPP was applied to grouping genes along Gene-Ontology categories or biological pathways (http://www.genmapp.org, Gladstone Institutes, University of California at San Francisco).

# References

## 1. Zhang X, Ren R (1998) Bcr-Abl efficiently induces a myeloproliferative disease and production of excess interleukin-3 and granulocyte-macrophage colony-stimulating factor in mice: a novel model for chronic myelogenous leukemia. Blood 92: 3829-3840.

## 2. Stojadinovic S, Low DA, Vicic M, Mutic S, Deasy JO, et al. (2006) Progress toward a microradiation therapy small animal conformal irradiator. Med Phys 33: 3834-3845.

## 3. de Guzman Strong C, Wertz PW, Wang C, Yang F, Meltzer PS, et al. (2006) Lipid defect underlies selective skin barrier impairment of an epidermal-specific deletion of Gata-3. J Cell Biol 175: 661-670.

## 
